# Motility-based label-free detection of parasites in bodily fluids using holographic speckle analysis and deep learning

**DOI:** 10.1038/s41377-018-0110-1

**Published:** 2018-12-12

**Authors:** Yibo Zhang, Hatice Ceylan Koydemir, Michelle M. Shimogawa, Sener Yalcin, Alexander Guziak, Tairan Liu, Ilker Oguz, Yujia Huang, Bijie Bai, Yilin Luo, Yi Luo, Zhensong Wei, Hongda Wang, Vittorio Bianco, Bohan Zhang, Rohan Nadkarni, Kent Hill, Aydogan Ozcan

**Affiliations:** 1 Electrical and Computer Engineering Department, University of California, Los Angeles, CA 90095 USA; 2 Bioengineering Department, University of California, Los Angeles, CA 90095 USA; 3 California NanoSystems Institute, University of California, Los Angeles, CA 90095 USA; 4 Department of Microbiology, Immunology, and Molecular Genetics, University of California, Los Angeles, CA 90095 USA; 5 Department of Physics and Astronomy, University of California, Los Angeles, CA 90095 USA; 6 Molecular Biology Institute, University of California, Los Angeles, CA 90095 USA; 7 Department of Surgery, David Geffen School of Medicine, University of California, Los Angeles, CA 90095 USA

## Abstract

Parasitic infections constitute a major global public health issue. Existing screening methods that are based on manual microscopic examination often struggle to provide sufficient volumetric throughput and sensitivity to facilitate early diagnosis. Here, we demonstrate a motility-based label-free computational imaging platform to rapidly detect motile parasites in optically dense bodily fluids by utilizing the locomotion of the parasites as a specific biomarker and endogenous contrast mechanism. Based on this principle, a cost-effective and mobile instrument, which rapidly screens ~3.2 mL of fluid sample in three dimensions, was built to automatically detect and count motile microorganisms using their holographic time-lapse speckle patterns. We demonstrate the capabilities of our platform by detecting trypanosomes, which are motile protozoan parasites, with various species that cause deadly diseases affecting millions of people worldwide. Using a holographic speckle analysis algorithm combined with deep learning-based classification, we demonstrate sensitive and label-free detection of trypanosomes within spiked whole blood and artificial cerebrospinal fluid (CSF) samples, achieving a limit of detection of ten trypanosomes per mL of whole blood (~five-fold better than the current state-of-the-art parasitological method) and three trypanosomes per mL of CSF. We further demonstrate that this platform can be applied to detect other motile parasites by imaging *Trichomonas vaginalis*, the causative agent of trichomoniasis, which affects 275 million people worldwide. With its cost-effective, portable design and rapid screening time, this unique platform has the potential to be applied for sensitive and timely diagnosis of neglected tropical diseases caused by motile parasites and other parasitic infections in resource-limited regions.

## Imaging: Detecting parasites with holograms and AI

Scientists have developed a cost-effective and portable technology that uses three-dimensional holograms to detect parasites in human bodily fluids, providing a more sensitive technique for diagnosing diseases, without the need for chemical labeling. A major global health problem, parasitic infections affect billions of people around the world and can lead to severe illnesses and death. Some of the current screening techniques, however, use manual microscopic examinations that struggle to provide the sensitivity required for early diagnoses. Now, Aydogan Ozcan and colleagues from the University of California Los Angeles in the United States have developed a cost-effective and portable optical device that uses lensless time-resolved holographic speckle imaging combined with artificial intelligence to automatically detect rare parasites in bodily fluids. The device has the potential to perform rapid and early diagnoses of diseases caused by parasitic infections and could be used in developing countries.

## Introduction

Parasitic infections affect billions of people globally and cause a massive socioeconomic burden^1–4^. Although they are usually associated with low-income countries, parasitic infections are becoming an increasing health concern in developed countries. In the United States, millions of people are affected by various parasites, which can cause severe illnesses and even death^1^. Motility is common among disease-causing organisms, from unicellular pathogenic bacteria and parasitic protozoa to multicellular parasitic worms and ectoparasites. The ability of an organism to move from one location to another location has distinct benefits for successful infection and transmission, and motility is often central to virulence^5–7^. Despite the importance of motility for a parasitic lifestyle, parasite motility remains an understudied area of research, and motility-based diagnostics are considerably underexplored.

Human African trypanosomiasis (HAT), which is also known as sleeping sickness, and Chagas disease (i.e., American trypanosomiasis) are examples of neglected tropical diseases caused by motile protozoan parasites. Neglected tropical diseases have historically been given little attention, disproportionately affect the world’s poorest people, and lack adequate medical interventions for diagnosis and treatment. Vaccines for these diseases do not exist, and existing chemotherapeutics suffer from high toxicity and drug resistance^8–11^. Two devastating diseases—HAT and Chagas disease—are caused by related trypanosome parasites^12^. *Trypanosoma brucei* (*T. brucei gambiense and T. brucei rhodesiense* subspecies) is responsible for HAT^9,10^, and related species cause animal diseases that present a substantial economic burden in some of the poorest areas of the world. The parasite is transmitted to humans by the tsetse fly and survives extracellularly in blood and tissues, with dramatic impacts on the central nervous system^9,10^. HAT is endemic in ~30 sub-Saharan Africa countries with ~65 million people who are at risk of infection^4^. The number of reported cases has decreased to historic lows but past declines in case numbers have been followed by major epidemics. Therefore, HAT remains an important human health risk^13^. Chagas disease is caused by *Trypanosoma cruzi* (*T. cruzi*), which invades and replicates inside host cells and causes severe pathology within host tissues^14^. Although Chagas disease is primarily transmitted by the bite of triatomine bugs, other transmission routes include blood transfusion and ingestion of contaminated food or drink^15^. The disease is endemic in Latin America, where it affects more than 6 million people^11,15,16^. More than 300,000 people in the United States are estimated to be infected^17^ with further increases expected as globalization and climate change impact the distribution of disease-transmitting vectors.

Both trypanosomiases can be classified into an initial stage during which trypanosomes circulate in the bloodstream and medical treatment is most effective (stage I HAT and acute Chagas disease) and a later stage that is exceedingly more difficult, if not impossible, to cure (stage II HAT and chronic Chagas disease). Therefore, early detection is crucial for both diseases. However, rapid and sensitive diagnosis remains challenging, particularly in resource-limited settings^18^. In the diagnosis of HAT, assessing the stage of the disease is essential to determine the appropriate therapeutic strategy. While trypanosomes remain in the blood and lymph in stage I HAT, stage II HAT is characterized by trypanosomes that cross the blood-brain barrier and invade the central nervous system, which causes neurological symptoms and eventually death if untreated. Because the drugs used to treat stage I and stage II are not interchangeable and the drugs for stage II may be more toxic, identification of the stage of the disease is critical to inform the selection of a treatment regimen. Stage determination is currently performed by collecting cerebrospinal fluid (CSF) via a lumbar puncture and examining the CSF under a microscope for the presence of white blood cells (WBCs) and trypanosomes.

The length and width of both trypanosome species are typically ~20 μm and ~3 μm, respectively, and both trypanosome species use flagellum-mediated motility for parasite propulsion^9,19,20^. Detection of these motile parasites in large-volume bodily fluids, such as blood and CSF, is an important clinical challenge. For decades, the standard screening test for *T. b. gambiense* HAT has been the card agglutination test for trypanosomiasis (CATT), which detects the presence of antibodies against a specific parasite antigen^4^. However, CATT suffers from practical limitations as well as low specificity and sensitivity in some areas^4^. Moreover, a positive CATT test must typically be confirmed with direct visual observation in blood samples. Several molecular and immunological detection methods, including polymerase chain reaction (PCR) and rapid diagnostic tests (RDTs), have been developed. However, these methods are limited by insufficient specificity or sensitivity, the need for sophisticated equipment and highly trained personnel, or high production costs^4,21,22^. Thus, microscopic evaluation remains extensively employed for primary or secondary diagnosis, and direct observation of CSF remains the sole method for HAT stage determination^4,22^. Each milliliter of whole blood typically contains billions of red blood cells (RBCs), millions of WBCs and hundreds of millions of platelets. Conversely, blood parasitemia fluctuates during the course of infection and often is less than 100 parasites/mL^18,23,24^, which renders microscopic identification of trypanosomes a needle-in-a-haystack problem. The low sensitivity of direct observation methods therefore requires analytical separation devices, such as centrifugation or ion exchange purification, which partially limit analysis in resource-limited settings^25–27^. Thus, the need for development of new methods with high sensitivity and throughput that can reduce costs and simplify diagnosis is urgent.

**Fig. 1:**
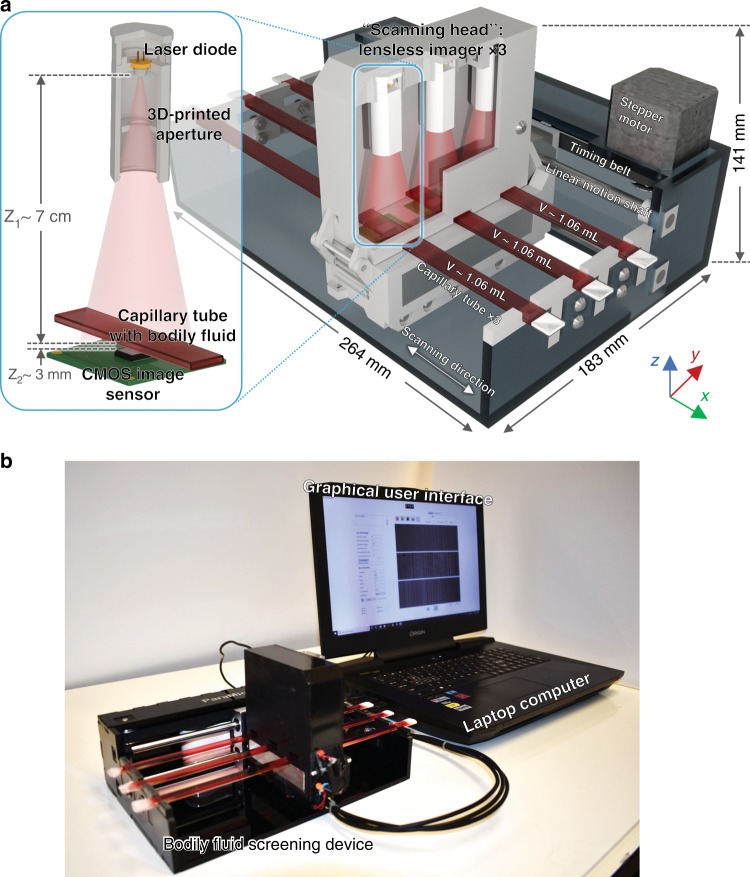
High-throughput bodily fluid screening device, which screens and analyzes ~3.2 mL of fluid sample within ~20 min. **a** Schematic of the device based on lensless holographic time-resolved speckle imaging. **b** Photograph of the device, controlled by a laptop, which is also used for processing the acquired data

**Fig. 2:**
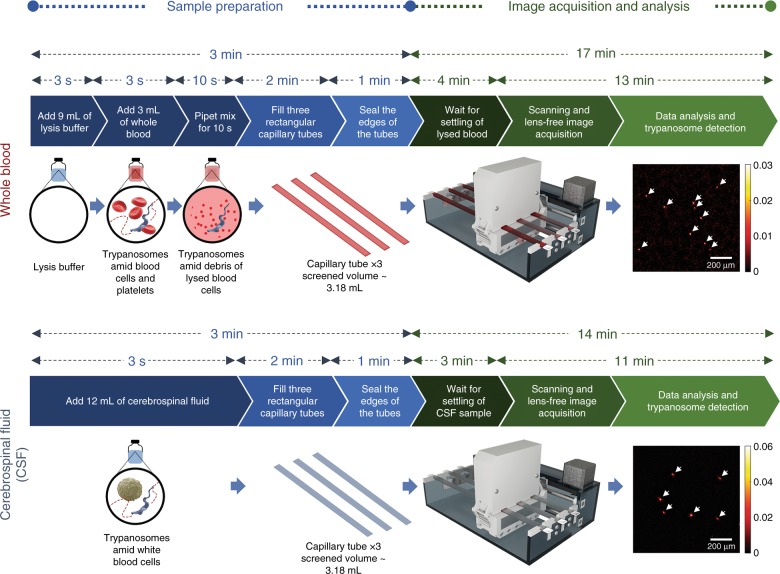
**Sample preparation and imaging process**

**Fig. 3:**
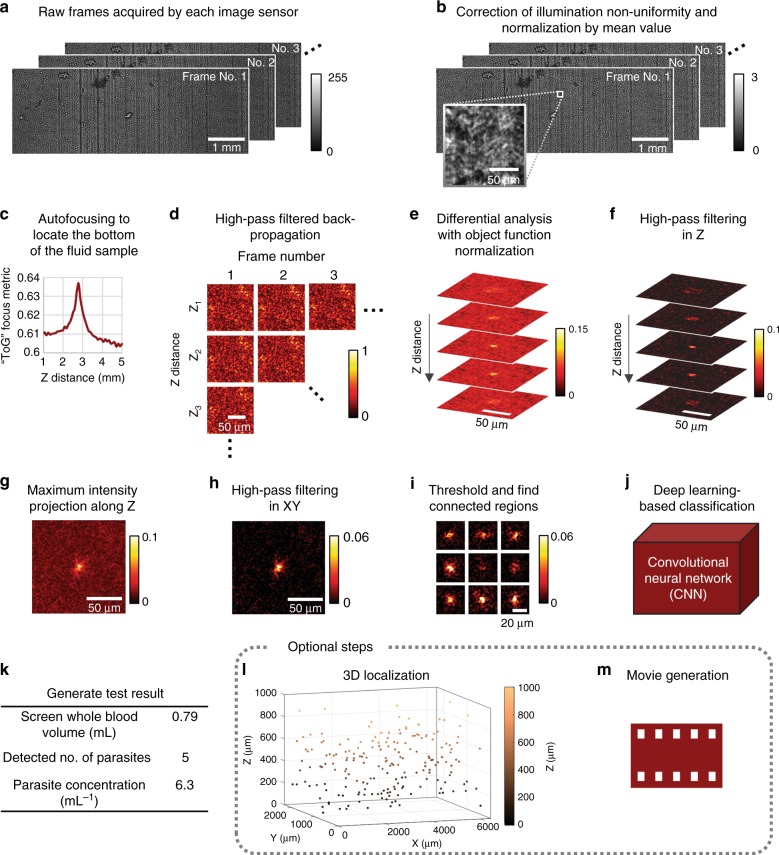
Image processing steps. Raw holographic speckle patterns are processed by the CMA algorithm with OFN, followed by deep learning-based identification, for sensitive and label-free detection of trypanosomes in lysed blood. The steps are performed in the same order as listed here

To address this important challenge, we demonstrate a cost-effective and field-portable optical device (Fig. 1), which is based on lensless time-resolved holographic speckle imaging, for label-free, high-throughput, and sensitive detection of motile parasites in various bodily fluids and turbid media. Instead of staining a target analyte or using molecular biomarkers, our technique utilizes the locomotion of self-propelling parasites (or other motile microorganisms) as a biomarker and endogenous contrast mechanism. As a result, the sample preparation is very simple and fast, and does not require any benchtop-scale sample processing device/equipment, refrigeration, centrifugation, or purification (Fig. 2). As shown in Fig. 1a, the fluid sample to be screened is illuminated by a coherent light source (e.g., a laser diode), and a complementary metal-oxide-semiconductor (CMOS) image sensor is placed below the sample to record the time-varying holographic speckle patterns of the sample. This image sequence is analyzed by a custom-written computational motion analysis (CMA) algorithm that is based on holography to generate a three-dimensional (3D) contrast map, which is specific to the locomotion of the parasites in the sample volume. A deep learning-based classifier is used to automatically detect and count the signal patterns of the parasites using the reconstructed 3D locomotion map (Fig. 3).

To increase the throughput and reduce the limit of detection (LoD) for rapid screening of large fluid volumes (~3.2 mL), we constructed a prototype that consists of three identical lensless speckle imaging modules mounted on a linear translation stage to screen three individual sample tubes in parallel (Fig. 1a and Supplementary Movie 1). Each imaging module is translated to different sections of the capillary tube that contains the liquid sample, where the CMOS image sensor captures high-frame-rate video sequences before advancing to the next section. Using this approach, ~3.2 mL of fluid sample is prepared, screened and analyzed within ~20 min using the setup shown in Fig. 1. Compared with standard benchtop optical microscopes, our design provides orders of magnitude increase in the screened sample volume (which is very important for the detection of parasites at low concentrations) and is significantly more compact and lightweight (1.69 kg). Furthermore, since a large sample volume is computationally screened in the axial direction, our imaging device does not need high precision in its opto-mechanical design, which also renders our platform highly cost-effective, where the total parts cost less than $1850 even in very low volume manufacturing.

We employed trypanosomes to test our mobile platform and demonstrated its capability to detect parasites in spiked whole blood and CSF samples (Fig. 2), which are important for the diagnosis and stage determination of HAT and the diagnosis of acute Chagas disease. We performed spiking experiments for a series of concentrations using *T. brucei brucei* (a nonhuman infectious subspecies of *Trypanosoma*) as a model parasite for *T.b. gambiense*, *T.b. rhodesiense* and *T. cruzi*. Via deep learning-based classification, we showed that as few as 10 parasites per mL of whole blood and 3 parasites per mL of CSF can be reliably detected using our platform. Furthermore, we demonstrated the success of our platform to detect other motile parasites in bodily fluids by imaging *Trichomonas vaginalis* (*T. vaginalis*), the protozoan parasite responsible for trichomoniasis, which is the most common, nonviral sexually transmitted disease that affects 3.7 million people in the United States and 275 million worldwide^28^. We believe that this label-free, motility-based parasite detection platform can provide a cost-effective and portable approach for rapid and sensitive screening of trypanosomes and other motile parasites in resource-limited settings or as a high-throughput analytical research tool to study motile organisms in three dimensions.

## Results

### Detection of parasite locomotion in three dimensions using holographic speckle analysis

**Fig. 4:**
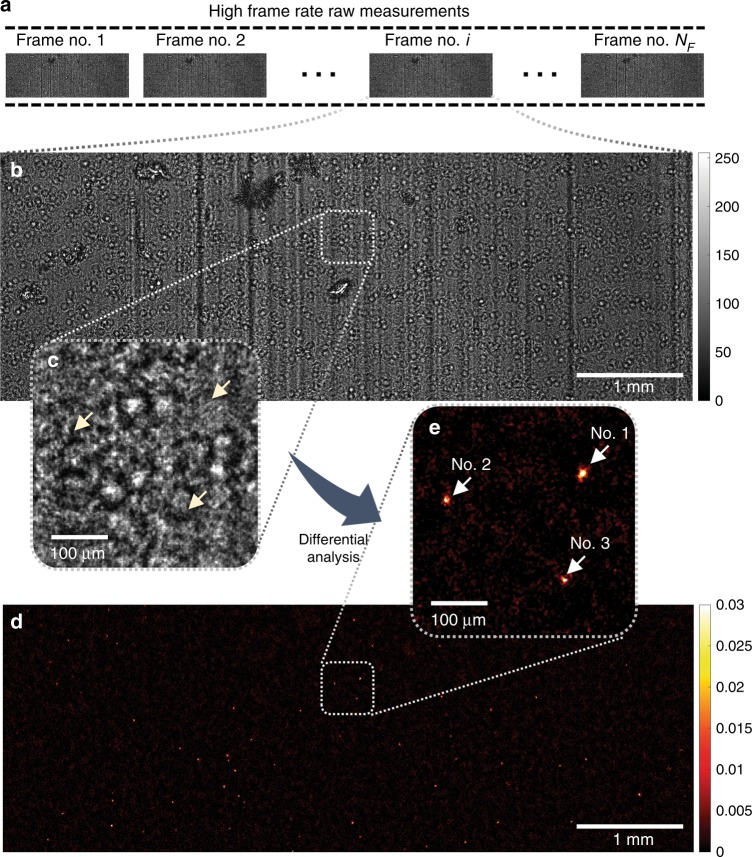
Experimental demonstration of applying the CMA algorithm and OFN to a lysed blood sample spiked with motile trypanosome parasites, over an FOV of ~14.7 mm^2^. **a** A time-sequence of the diffraction patterns of the sample is captured. **b, c** One of the frames in the raw image sequence is shown. The diffraction pattern is severely speckled due to the light scattering by the cell debris, which renders the parasites invisible (yellow arrows in (**c**)). **d**, **e** After being processed by the CMA algorithm, motile parasites can be detected. The amplitude and phase movies of the three trypanosomes in **e** (indicated by white arrows) are also shown in Supplementary Movie 2

To sustain a high frame rate (~26.6 fps), which is essential to our parasite detection technique, we chose to split the full field of view (FOV) of the image sensor in two halves, where each half ~14.7 mm^2^. Figure 4a-c shows the raw speckle patterns of a lysed, trypanosome-spiked whole blood sample captured by our image sensor. Even though this simple lysis process has significantly reduced the density of the blood sample, the interference patterns (e.g., Fig. 4b, c)) remain highly dense due to the random light scattering from the cell debris in the lysed blood. As a result, the diffraction patterns of the optically transparent and weakly scattering trypanosomes (Fig. 4c, yellow arrows) are buried under the speckle patterns, which renders their direct detection extremely challenging.

To address this challenge, the spatial-temporal variations in the detected speckle patterns due to the rapid locomotion of motile trypanosomes within blood can be utilized. We took advantage of this approach and developed a CMA algorithm, which involves holographic back-propagation, differential imaging (with an optimally adjusted frame interval for trypanosome locomotion), and temporal averaging, conducted at each horizontal cross-section within the sample volume. Object function normalization (OFN) was introduced into each differential imaging step to suppress potential false positives due to unwanted, strongly scattering objects within the sample. The algorithm was followed by post-image processing and deep learning-based classification to identify the signals caused by trypanosomes (refer to the Materials and Methods section for details). Figure 4d, e exemplify the results of this computational process, where the “hotspots” in the processed images correspond to motile trypanosomes. To better illustrate this, based on the calculated 3D locations of the three hotspots in Fig. 4e (indicated by white arrows), we created in-focus movies of the amplitude and phase channels of the back-propagated diffraction patterns (refer to Supplementary Movie 2). Rapid locomotion of these trypanosomes can be observed in this video, although they are partially obscured by the interference patterns created by the other nonmotile objects (e.g., cell debris) in the sample.

Similarly, the results of imaging trypanosomes within WBC-spiked artificial CSF samples are shown in Supplementary Fig. S1 and Supplementary Movie 3. Because CSF is predominantly a clear medium, the background noise level caused by quasi-static scatterers in the medium is significantly lower than the motile trypanosome signal level (i.e., the hotspots in Supplementary Fig. S1b). Digitally focused amplitude and phase movies also show lower-noise reconstructions of these motile trypanosomes, as shown in Supplementary Movie 3.

As detailed in Table 1, >80% of the total image processing time to image and detect these trypanosomes is spent on the CMA algorithm, which involves thousands of fast Fourier transforms of ~6-megapixel images for each recorded image sequence (refer to the Materials and Methods section for details). Therefore, graphics processing unit (GPU)-based parallel computing is essential for the speed-up of the CMA algorithm. Using a single GPU, the entire image processing task for one experiment (a total of 216 image sequences for the three parallel image sensors) requires ~26 min and ~21 min for a blood sample and a CSF sample, respectively. When using two GPUs, because each GPU is given a separate image sequence to process at a given time, there is minimal interference between the GPUs and maximal parallelism can be achieved. Therefore, ~2-fold speed-up is observed when using two GPUs, which results in a total image processing time of ~13 min and ~11 min for a blood sample and a CSF sample, respectively. Combined with all the other sample preparation steps, the total detection time per test is ~20 min and ~17 min for a blood sample and a CSF sample, respectively (refer to Fig. 2 for details).

**Table 1:** Image processing time

	Single GPU		Dual GPUs	
Processing step	Time per image sequence (ms) Blood/CSF	Total time per test (min) Blood/CSF	Time per image sequence (ms) Blood/CSF	Total time per test (min) Blood/CSF
Copy data from CPU memory to GPU memory	316.7/212.7	1.14/0.77	346.2/231.7	0.62/0.42
Image normalization	44.3/30.0	0.16/0.11	49.0/32.5	0.09/0.06
Autofocusing	523.0/NA	1.88/NA	680.8/NA	1.23/NA
Computational motion analysis	6205.0/5515.7	22.34/19.86	6137.8/5561.5	11.05/10.01
Post-image filtering	106.7/136.0	0.38/0.49	108.2/138.3	0.19/0.25
Segmentation	10.1/10.1	0.04/0.04	10.1/10.1	0.02/0.02
Deep learning-based classification	9.8/10.3	0.04/0.04	9.8/10.3	0.02/0.02
Total	7215.6/5914.8	25.98/21.29	7341.9/5984.4	13.22/10.77

### Quantification of the LoD for trypanosomes

**Fig. 5:**
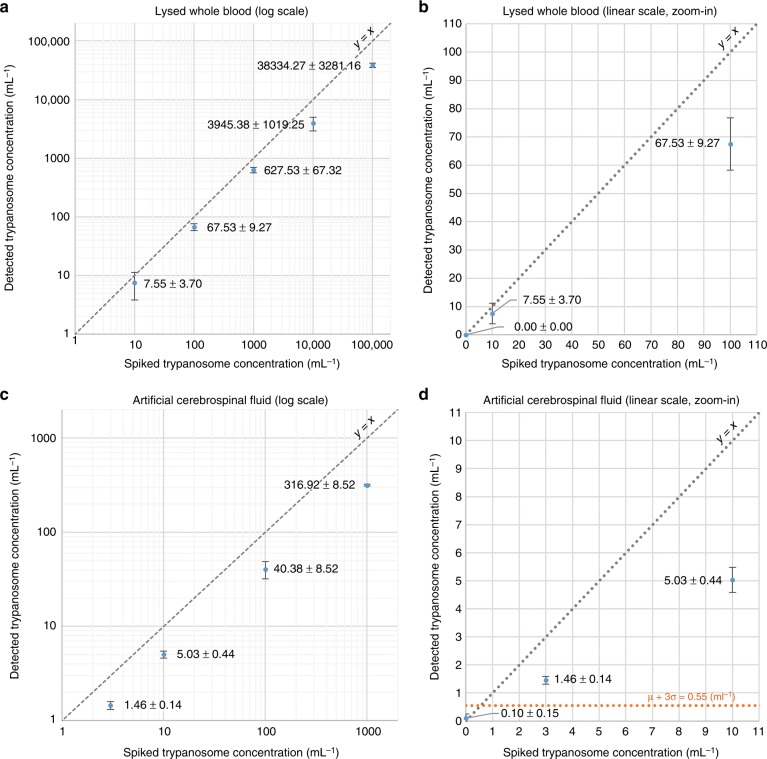
Quantification of the LoD of our platform for detecting trypanosomes in lysed whole blood and artificial CSF samples. **a** Calibration curve for trypanosome detection in lysed whole blood (logarithmic scale). Dashed line indicates expected values (*y* = *x*). Measured values are indicated by blue data points. Error bars show the standard deviation of 3 independent measurements. **b** Zoom-in of (**a**) shows the low concentration measurements (linear scale), including the negative control (no trypanosomes). No false positives were found in the three negative control samples. **c**, **d** Calibration curves for trypanosome detection in artificial CSF, similar to (**a**, **b**). Orange dashed line in **d** corresponds to the mean +3 × standard deviation of the negative control result

We determined the LoD of our platform for detecting trypanosomes in lysed whole blood by performing serial dilution experiments; the results are shown in Fig. 5a, b. We spiked trypanosome-infected mouse blood into uninfected blood to generate a series of parasite concentrations, including 0 mL^−1^ (negative control), 10 mL^−1^, 100 mL^−1^, 1000 mL^−1^, 10,000 mL^−1^ and 100,000 mL^−1^, where *N* = 3 replicate experiments were performed at each concentration. As shown in Fig. 5a, false positives were not detected in the three negative control samples, while our detected concentration was 7.55 ± 3.70 mL^−1^ for the three 10 mL^−1^ experiments. Therefore, we conclude that our LoD is ~10 trypanosomes per mL of whole blood, which is 5× better than the best currently available parasitological detection method (i.e., the mini anion exchange centrifugation technique, mAECT^25^; refer to Table 2). Figure 5 also reveals that the recovery rate (detected trypanosome concentration divided by the spiked concentration) of our technique ranges from ~68–76% (at the lower end of our tested concentrations) to ~38–39% (at the higher end of our tested concentrations). This concentration-dependent recovery rate is possibly related to the proximity of the trypanosomes to each other at higher concentrations, which produces more than one trypanosome in a 64 × 64-pixel cropped image that may be misclassified as negative by the deep learning-based classifier (refer to the Materials and Methods section for details) and causes underestimation of the true number of trypanosomes in the sample. This reduction in the recovery rate observed at high concentrations can be potentially compensated by calibration.

**Table 2:** Comparison of existing parasitological detection methods against our method

	Parasite	*T. cruzi*		*T. brucei gambiense*		*T. brucei rhodesiense*		Blood	CSF	LoD/Sensitivity/Specificity	Blood volume analyzed	CSF volume analyzed	Expert requirement (e.g., benchtop microscope)	Benchtop device requirement for sample preparation (e.g., centrifugation)	Electricity requirement	Field-portable	Microscopic examination of parasites manually	Total analysis time per test
	Stage	Acute	Chronic	I	II	I	II											
mAECT tubes^a 25^				✓	✓			✓		<50 trypanosomes/mL	350 μL/test		Yes	Yes	Yes	No	Yes	45–60 min
PCR^34^		✓	✓	✓^34^	✓^34^	✓^35^	✓^34^	✓	✓	High sensitivity and specificity for *T. brucei gambiense* and *T. cruzi*^36^ Low sensitivity for *T. brucei rhodesiense*^35^	200 μL^34^	1000 μL^34^	Yes	Yes	Yes	Yes	No	~5 h^b^
Thick smear^37^		✓	✓	✓	✓	✓	✓	✓	✓	Low sensitivity	2–4 μL/slide	2–4 μL/slide	Yes	Yes	Yes	No	Yes	75–90 min
Microhematocrit		✓^38^		✓^18^	✓^18^	✓^18^	✓^18^	✓^18^	✓^18^	High sensitivity for *T. b. gambiense* and *T.b. rhodesiense* (an estimated detection threshold of 500 trypanosomes/ml)^18^	6 × 50–75 μL/tube		Yes	Yes	Yes	No	Yes	~15 min^38^
Strout method^39^		✓		✓	✓	✓	✓	✓		Low sensitivity	2–4 μL/slide^c^		Yes	Yes	Yes	No	Yes	~30 min^d^
Card agglutination test for trypanosomiasis (CATT)^39^				✓	✓			✓		High specificity and sensitivity for *T.b. gambiense*^e^ Not for *T. cruzi* and *T.b. rhodesiense*	30–50 μL^40^		No	No	No^f^	Yes	No	5 min
Rapid diagnostic tests^4,40^		✓^41^		✓	✓			✓		Low specificity, especially for low parasitemia levels^4^	30 μL^42^		No	No	No	Yes	No	15 min^42^
***Our method***		✓		✓	✓	✓	✓	✓	✓	*10 trypanosomes/mL for blood; 3 trypanosomes/mL for CSF*	~*800* *μL*	~*3200* *μL*	*No*	*No*	*Yes*	*Yes*	*—*	~*20* *min*

The bold and italics text/values were used to emphasize the row that includes the specifications of our method

^a^This method can be made even more sensitive (10 trypanosomes/mL) by performing centrifugation of whole blood and loading buffy coat onto the mAECT tube^43^

^b^Including DNA isolation, PCR, and electrophoresis

^c^The blood analyzed (2–4 μL) is taken from a centrifuged/concentrated blood sample

^d^5 min for centrifugation plus time required to analyze each slide

^e^CATT does not work for some strains of *T. b. gambiense* because they lack the specific antigen; and its specificity is low in areas with low prevalence^4^

^f^CATT requires a rotator to mix the blood sample for 5 min at 60 rpm

For *T. brucei*, stage determination is critical for determining the most appropriate treatment regimen, which is currently performed by collecting CSF via a lumbar puncture and examining the CSF under a microscope. Patients with ≤5 WBCs per μL and no trypanosomes in their CSF are classified as stage I; otherwise, if there are >5 WBCs per μL or if trypanosomes are found in the CSF, they are classified as stage II (ref. 4). To address this need for high-throughput CSF screening, we also quantified the LoD of our platform to detect trypanosomes in CSF. For this purpose, we employed an artificial CSF^29^ sample that is spiked with human WBCs, where cultured trypanosomes were spiked in the artificial CSF solution at concentrations of 3 mL^−1^, 10 mL^−1^, 100 mL^−1^, and 1000 mL^−1^, in addition to a negative control (*N* = 3 for each concentration). The concentration of spiked human WBCs was selected as 20 WBCs/μL to evaluate the performance of the device to detect trypanosomes in a scenario where the WBC concentration was four times higher than the 5 μL^−1^ threshold in the stage determination. Unlike the blood sample, the CSF solution is optically clear, and lysis was not needed, which helped us to further improve our LoD: as shown in Fig. 5c, d, our platform was able to detect trypanosomes spiked in CSF with a very low LoD of 3 trypanosomes per mL.

### Detection of *T. vaginalis* parasites

Although we chose to validate our motility-based detection approach on *T. brucei*, we anticipate this approach to be broadly applicable for the detection of a variety of motile microorganisms. As a preliminary test of the performance of our device on a completely different motile parasite, we chose *T. vaginalis*, the protozoan parasite responsible for trichomoniasis, which is the most common nonviral sexually transmitted disease in the United States and worldwide^28,30–32^. *T vaginalis* infects the urogenital tract of both women and men. Although it is often asymptomatic, *T. vaginalis* infection has been associated with increased risk related to other health conditions, including human immunodeficiency virus infection, preterm labor, pelvic inflammatory disease, and prostate cancer^2,31^. For the diagnosis of trichomoniasis, cell culture followed by microscopy remains the gold standard, which is highly sensitive and can detect *T. vaginalis* from an inoculum that contains as few as three parasites per mL^30^. However, it is limited by high cost, inconvenience, and lengthy examination time, as well as susceptibility to sample contamination. The most common diagnostic method—wet-mount microscopy—suffers from poor sensitivity (51–65%)^32,33^. Thus, our highly sensitive lensless time-resolved holographic speckle imaging method can provide substantial benefit.

With only minor adjustments to our algorithm (refer to the Materials and Methods section), we demonstrated that our platform can detect *T. vaginalis* in phosphate-buffered saline (PBS) solution and culture medium (refer to Supplementary Figs. S2 and S3 and Supplementary Movies 4 and 5). Based on these experiments, we observe that *T. vaginalis* creates significantly stronger signal intensities compared with trypanosomes in CSF (Supplementary Fig. S1), which is related to the intense locomotion and strong light scattering of *T. vaginalis*. This finding suggests that our platform can be potentially applied to achieve a similar, if not better, sensitivity level for *T. vaginalis*, e.g., attaining ≤ 3 parasites per mL. Additional testing is needed to establish the LoD of *T. vaginalis* from different environments, such as culture medium and urine, which corresponds to different clinical needs, such as the detection of *T. vaginalis* from diluted vaginal secretion or direct detection from urine.

## Discussion

We presented a new method of motility-based parasite detection, which is based on lensless time-resolved holographic speckle imaging, and demonstrated that it is effective for rapid detection of trypanosomes within lysed blood and CSF, achieving an LoD that is better than the LoD of current parasitological methods (refer to Fig. 5 and Table 2)^4,18,25,34–43^. This automated technique has the potential to improve parasite screening efficiency while reducing the need for highly specialized and expensive equipment and expertise that are essential to PCR-based or microscopic detection methods. The total cost for all parts of our instrument, excluding the laptop, is less than $1850; this cost can be easily reduced to $500–1000 in large-volume manufacturing. Our total analysis time, including all the sample preparation steps, is only ~20 min, which is comparable to or faster than most existing methods (Table 2). This motility-based method achieves high sensitivity without requiring specialized detection reagents, refrigeration, centrifugation, or purification, which renders it more versatile for the analysis of different types of samples (e.g., blood and CSF) and less susceptible to differences between parasite subspecies or isolates from different regions of the world. Therefore, the presented prototype can be readily adapted to any established or mobile clinic with access to electricity or battery power, which represents an advancement that can be a useful addition to existing diagnostic methods.

This diagnostic method can also be beneficial for improving the diagnosis of bloodstream HAT or Chagas infection or facilitating earlier identification of stage II HAT cases, when the parasitemia in the CSF is under the LoD of traditional methods and when the WBCs in the CSF remain scarce. This platform may also be useful for follow-up after disease treatment to screen patients for earlier and more sensitive detection of relapse. These advances can produce improved treatment outcomes for patients and increase the cure rate of disease. In addition to HAT, animal trypanosomiasis severely limits economic development. Therefore, applying motility-based detection to aid the screening of infected livestock and development of vector control options can help to reduce poverty in endemic areas^44^. In the case of Chagas disease, this technique can be adapted for screening blood donors or blood products, as well as sugarcane juice and acai juice products to help reduce various routes of transmission. Given the large populations at risk, the ability to rapidly analyze various types of samples/liquids in a simple and automated fashion will be particularly critical for developing a viable strategy to screen samples in regions where disease incidence declines due to eradication efforts.

While we employed three lensless imaging modules in our device to increase the throughput of our platform, our design can be easily modified to incorporate other numbers of parallel imaging channels for different applications and requirements, in which each additional channel offers ~1.06 mL of extra screening volume (or ~0.265 mL of whole blood volume). Depending on the application, these multiple imaging channels can be used to screen the bodily fluid sample of the same patient, which can increase the probability of detecting parasites at even lower concentrations. Or alternatively, the imaging channels can also be used to simultaneously screen samples of different patients to increase the throughput. Certainly, scaling up the number of lensless speckle imaging modules will increase the cost, dimensions and weight of the device. The cost will increase approximately linearly as a function of the number of imaging channels. This upscaling may also require modifying the linear motion system due to the increased weight of the scanning head, e.g., by arranging linear motion shafts/rails on both sides of the scanning head to balance the weight. With larger dimensions and heavier weights, the portability of the system will be compromised, which may be an important feature for users, especially for resource-limited settings. Additionally, a faster data interface and stronger computational power will also be needed for the controlling computer to timely transfer and process the increased amount of data.

Our device and label-free detection method takes advantage of the locomotion patterns of parasites to maximize the detection signal-to-noise ratio (SNR). Trypanosomes are known for their incessant motion, and motility is crucial to their survival and virulence in the host^7^. The swimming behavior of trypanosomes is highly complex^19,45^. Because the flagellum is laterally attached to the cell body, parasite translocation is accompanied by cell body rotation, which produces a “corkscrew” swimming pattern. In addition to cell translocation, the flagellum generates rapid, three-dimensional beating patterns. The average beating frequency of *T. brucei* is estimated to be 18.3 ± 2.5 Hz in forward-moving cells and 13.1 ± 0.8 Hz in backward-moving cells, whereas the rotational frequency of forward-moving cells is 2.8 ± 0.4 Hz^46^. The frame rate that matches the average beating frequency (forward-moving), according to the Nyquist sampling rate, is equal to 36.6 fps. In other words, a frame rate of at least 36.6 fps is able to record the speckle changes that correspond to each flagellar stroke. Even higher frame rates can record the speckle changes with finer time resolution, which corresponds to different time points during a flagellar stroke. Assuming an optimal subtraction time interval (Δ*t*) and time window (*T*) (refer to Materials and Methods, Supplementary Methods and Supplementary Fig. S4), a higher frame rate generates richer time-resolved information about speckle changes induced by motile parasites and additional frames that can be used for averaging, which can improve the overall SNR. However, because our goal is to generate contrast based on locomotion rather than a high-fidelity recording of the beating patterns, frame rates that are less than 36.6 fps are also acceptable for detection purposes. Considering the scanning time and the amount of acquired data, we chose to use a frame rate of ~26.6 fps for our platform. For future versions of the platform, faster image sensors and data interfaces can be employed to achieve higher frame rates, which can improve the SNR without increasing the data acquisition time.

*T.b. brucei* is extensively employed as a model microorganism for the study of trypanosomes because it is nonpathogenic to humans and therefore safe to conduct experiments on. We anticipate that this approach will be readily applicable to *T.b. gambiense*, *T.b. rhodesiense* and *T. cruzi* since their movements are fundamentally similar; however, direct testing on human-infectious trypanosomes is needed. Mouse blood and an artificial CSF solution were utilized throughout our testing due to safety concerns but our lysis buffer also works with human blood. Future research will focus on testing patient samples from endemic regions to establish the sensitivity and specificity of our presented technique for the diagnosis of various trypanosomiases.

Numerous motile organisms can cause infections in humans^5,6,47^. The current state of our technique does not automatically differentiate different parasites. However, the amplitude and phase movie that is generated for each detected signal (Fig. 3m and Supplementary Movies 2-5) can potentially enable a trained clinician to distinguish different motile parasites based on the morphology, size, and motility pattern, which is analogous to observing each live parasite under a brightfield and phase-contrast microscope. The prevalence of particular pathogens in the region can also aid in this regard. Furthermore, a trained video classifier that is based on, e.g., a convolutional neural network (CNN) or a recursive neural network (RNN), can potentially distinguish and automatically identify various parasites, if sufficient training data are provided.

In this study, we utilized trypanosomes to demonstrate the feasibility of employing lensless time-resolved holographic speckle imaging in the detection of parasitic infection. While our approach capitalized on the motility of trypanosomes, this platform is broadly applicable to other motile parasites, including other eukaryotic parasites, such as *T. vaginalis* (refer to Supplementary Figs. S2 and S3; and Supplementary Movies 4 and 5) and other fluid samples beyond those tested in this study. In principle, this platform can also be used for the detection of *Loa loa* (*L. loa*) microfilariae in blood samples, which are significantly larger (~0.2–0.3 mm in length) than the parasites investigated in this paper. For these large motile parasites, as an alternative approach, D’Ambrosio et al. used a cellphone-based detection method by taking advantage of the displacement of the RBCs caused by the collision with *L. loa* microfilariae in an imaging chamber^48^. This design is compact and cost-effective; however, it suffers from a considerably smaller detection volume (~0.01 mL) than that in our method, which screens and automatically processes ~0.8 mL of whole blood or ~3.2 mL of CSF. Furthermore, it would be very challenging for this earlier cellphone-based design to be used for the detection of parasitic protozoa, such as trypanosomes, which have more than an order-of-magnitude smaller size and mass and lower parasitemia, as well as substantially weaker light scattering than that of *L. loa*.

Motile bacteria also cause numerous human diseases^6^. Although bacteria are typically considerably smaller than trypanosomes, the concept of motility-based detection combined with optical magnification can also be explored for label-free detection of bacterial pathogens. Potential uses of motility-based detection for screening of other bodily fluids, such as urine or diluted mucosal secretions and stool samples, may exist. Therefore, we believe that this presented approach has considerable potential to impact various global health challenges. Additionally, we believe that using motility as a biomarker and endogenous contrast can create new possibilities beyond clinical diagnostics. As a label-free 3D imaging modality that is robust to light scattering and optically dense media, this approach can potentially be employed to study motile microorganisms within various fluid environments in a high-throughput manner and benefit biomedical research. As another potential application, a speckle decorrelation approach that is similar to the approach demonstrated by Mandracchia et al.^49^, may be adopted for our platform to measure, e.g., viability of probiotic bacteria in different environments, which may be useful for, e.g., the food industry.

## Materials and methods

### Sample preparation

#### Lysis buffer preparation

A volume of 44 mM sodium chloride (product No. 71379, Sigma Aldrich (Missouri, USA)), 57 mM disodium phosphate (product No. 30412, Sigma Aldrich, (Missouri, USA)), 3 mM monopotassium phosphate (product No. 60220, Sigma Aldrich, (Missouri, USA)), 55 mM glucose (product No. G8270, Sigma Aldrich, (Missouri, USA)), and 0.24% (w/v) sodium dodecyl sulfate (product No. L4390, Sigma Aldrich, (Missouri, USA)) in reagent grade water (product No. 23-249-581, Fisher Scientific (New Hampshire, USA)) were mixed for 2 h using a magnetic stir bar on a magnetic mixer. The solution was filtered using a disposable filtration unit (product No. 09-740-65B, Fisher Scientific, (New Hampshire, USA)) for sterilization and was stored at room temperature. This buffer solution lyses all components of whole blood, including RBCs, and WBCs, but does not lyse the trypanosomes^50^.

#### Artificial CSF preparation

According to a previous method^29^, 1.25 M sodium chloride, 260 mM sodium bicarbonate (product No. SX0320-1, EMD Millipore, (Massachusetts, USA)), 12.5 mM sodium phosphate monobasic (product No. S6566, Sigma Aldrich), and 25 mM potassium chloride (product No. P5405, Sigma Aldrich, (Missouri, USA)) were well mixed, and 10 mM magnesium chloride (product No. 208337, Sigma Aldrich, (Missouri, USA)) was added to produce 10X artificial CSF. The solution was filtered using a disposable filtration unit for sterilization. The 10X stock solution was diluted ten-fold with reagent grade water to make 1X artificial CSF^29^.

#### Culturing trypanosomes

427-derived bloodstream single marker trypanosomes (*T. b. brucei*) were cultivated at 37 °C with 5% CO_2_ in HMI-9 medium with 10% heat-inactivated fetal bovine serum (product No. 10438026, Gibco (Massachusetts, USA)) as described in ref. 51.

#### Collection of trypanosome-infected mouse blood

All the experiments that involve mice were carried out in accordance with the guidelines and regulations of the UCLA Institutional Animal Care and Use Committee (IACUC), NIH Public Health Service Policy on Humane Care and Use of Animals, USDA Animal Welfare regulations, and AAALAC International accreditation standards under IACUC-approved protocol ARC# 2001-065. Mouse infections were performed as described in ref. 52 with the following modifications: female BALB/cJ mice (product No. 000651, Jackson Laboratory, age 11–24 weeks, (Maine, USA)) were intraperitoneally injected with 5×10^5^-1×10^6^ parasites in 0.1–0.2 mL ice-cold phosphate-buffered saline with 1% glucose (PBS-G). Parasitemia was monitored by counting in a hemocytometer, and infected blood samples were collected when parasitemia reached ~10^7^–10^8^ parasites/mL. Infected blood was collected from either the saphenous vein or by cardiac puncture after euthanasia into heparinized capillary tubes (product No. 22-260950, Fisher Scientific, (New Hampshire, USA)) or heparinized collection tubes (product No. 8881320256, Covidien (Dublin, Republic of Ireland)).

#### Separation of WBCs from human blood

We used Ficoll-Paque PREMIUM (product No. 45-001-751, Fisher Scientific, (New Hampshire, USA)) for in vitro isolation of mononuclear cells from blood using density gradient separation according to manufacturer’s instructions. Human blood samples were acquired from UCLA Blood and Platelet Center after deidentification of patients and related information and were utilized in the separation of WBCs from blood. We mixed 2 mL ethylenediaminetetraacetic acid (EDTA)-treated blood with 2 mL sterile PBS (product No. 10-010-049, Fisher Scientific, (New Hampshire, USA)) in a 5 mL centrifuge tube (product No. 14-282-300, Fisher Scientific, (New Hampshire, USA)) by drawing the mixture in and out of a pipette. We put 3 mL of Ficoll-Paque PREMIUM in a 15 mL conical centrifuge tube (product No. 14-959-53A, Fisher Scientific, (New Hampshire, USA)) and carefully layered the diluted blood sample on the Ficoll-Paque PREMIUM. The suspension was centrifuged at 400× *g* for 40 min at 19 °C using a centrifuge with swing-out rotors (Allegra X-22R, Beckman-Coulter (California, USA)). After centrifugation, the upper layer, which contains plasma and platelets, was removed and mononuclear cells were transferred to a sterile centrifuge tube. To wash the cell isolate, it was mixed in 6 mL PBS and centrifuged at 400× *g* at 19 °C for 13 min The washing step was repeated twice, and the pellet was suspended in 1 mL PBS. The concentration of WBC was determined by counting in a hemocytometer and diluted to a stock solution of 8 × 10^5^ WBC/mL in PBS.

#### Protocol for calibration curve analysis for blood samples

Freshly collected trypanosome-infected mouse blood was diluted in uninfected mouse blood (Balb/C, female, pooled, sodium heparin, Charles River Inc. (Massachusetts, USA)) to a concentration of approximately 10^6^ parasites/mL. A sample of this trypanosome-infected blood was lysed with 3 volumes of lysis buffer, and the trypanosome concentration was determined by counting in a hemocytometer. The trypanosome-infected blood was diluted with uninfected blood to achieve the desired concentrations for calibration curve analysis.

#### Protocol for calibration curve analysis for CSF samples

Cultured trypanosomes were freshly harvested for each measurement to ensure consistent parasite motility. Trypanosomes were grown to a concentration of ~1 × 10^6^–1.5 × 10^6^ cells/mL and harvested by centrifugation at 1200× *g* for 5 min. The cell pellet was resuspended in 1 mL of PBS-G and diluted ~10-fold to 10^5^ cells/mL in PBS-G. The trypanosome concentration was determined by counting in a hemocytometer and the sample was diluted into 1X artificial CSF to achieve the desired concentrations for calibration curve analysis.

#### Sample preparation for imaging

We performed experiments using blood and artificial CSF samples. Borosilicate capillary tubes (inner dimensions: 1 mm height × 10 mm width × ~30 cm length; product No. LRT-1-10-67, Friedrich & Dimmock, Inc. (New Jersey, USA)) were prepared by dipping one end of the capillary tube into Vaseline jelly to plug the end. Plastic capillaries, e.g., capillaries constructed of acrylic, can also be utilized instead of glass. Excess jelly was removed using a Kimwipe (product No. 06-666, Fisher Scientific, (New Hampshire, USA)), and the tube end was sealed with parafilm (product No. 13-374-12, Fisher Scientific, (New Hampshire, USA)). For each tube, we prepared 4 mL samples. For blood samples, 3 mL of lysis buffer was mixed with 1 mL of uninfected or infected whole blood in a centrifuge tube. For CSF samples, we placed 100 μL WBC stock solution into trypanosome-infected artificial CSF to have 2 × 10^4^ WBCs/mL (i.e., 20 WBCs/μL) in the final mixture. Each sample was well mixed by drawing the mixture in and out of a pipette before loading into the capillary tube. The open end of the capillary tube was sealed using the jelly and parafilm. The glass capillary was cleaned using a Kimwipe that was moistened with methanol (product No. A452SK-4, Fisher Scientific, (New Hampshire, USA)) and installed on the device.

#### Culturing *T. vaginalis*

*T. vaginalis* strain G3 (Beckenham, UK 1973, ATCC-PRA-98) was cultured in modified  Diamond's trypticase-yeast extract-maltose (TYM) media that was supplemented with 10% horse serum (Sigma Aldrich (Missouri, USA)), 10 U/ml penicillin–10 μg/ml streptomycin (Invitrogen (California, USA)), 180 μM ferrous ammonium sulfate, and 28 µM sulfosalicylic acid at 37 °C^53^. The culture was passaged daily and maintained at an approximate concentration of 1 × 10^6^ cells/mL.

### Design of the high-throughput lensless time-resolved speckle imaging platform

As shown in Fig. 1a, the device consists of five main modules: (1) “scanning head” that consists of three lensless holographic speckle imagers, (2) linear translation stage, (3) housing, (4) circuit, and (5) control program. Each module is detailed in this section.

1. Scanning head: Three identical lensless imagers are built next to each other and housed by 3D-printed plastic using a 3D printer (Objet30 Pro, Stratasys (Minnesota, USA)). As shown in the inset of Fig. 1a, each lensless imager employs a 650-nm laser diode (product No. AML-N056-650001-01, Arima Lasers Corp. (Taoyuan, Taiwan, China)) as the illumination source, which has an output power of ~1 mW. The emitted light is passed through a 3D-printed aperture to limit its emission angle and avoid light leakage into the adjacent imagers. The sample, which is a glass capillary tube filled with the bodily fluid to be screened, is placed ~7 cm (z_1_ distance) below the laser diode. The sample’s diffraction pattern is imaged by a 10-megapixel CMOS image sensor (product No. acA3800-14um, Basler (Ahrensburg, Germany)) with a 1.67 μm pixel size and an active area of 6.4 mm × 4.6 mm (29.4 mm^2^), which is placed below the capillary tube. The air gap between the image sensor and the bottom surface of the glass capillary tube ranges from ~1–1.5 mm to reduce the heat transfer from the image sensor to the sample. Because each image sensor has multiple circuit boards that generate heat, custom-made aluminum heat sinks are inserted between the circuit boards and arranged on the sides of the scanning head to dissipate heat and prevent image sensor malfunction.
2. Linear translation stage: A linear translation stage is built from two linear motion shafts (product No. 85421, Makeblock Co., Ltd. (Shenzhen, China)), two linear motion sliders (product No. 86050, Makeblock Co., Ltd. (Shenzhen, China)), a timing belt (product No. B375-210XL, ServoCity (Kansas, USA)), two timing pulleys (product No. 615418, ServoCity (Kansas, USA)), and a stepper motor (product No. 324, Adafruit Industries LLC. (New York, USA)). The scanning head is mounted onto the sliders using screws.
3. Housing: The housing of the scanning head is constructed with 3D-printed plastic. The outer shell of the device is constructed with laser-cut ¼-inch acrylic sheets.
4. Circuit: A printed circuit board (PCB) is custom-built to automate the device, which contains a microcontroller (Teensy LC, PJRC (Oregon, USA)) connected to the laptop computer via USB 2.0, laser diode driver circuits built from constant current circuits (product No. LM317DCYR, Texas Instruments (Texas, USA)), and a stepper motor driver circuit (product No. TB6612, Adafruit). The laser diodes and the stepper motor are powered using a 12 V power adapter. Various digital switches built from metal-oxide-semiconductor field-effect transistors (MOSFETs) are controlled by the digital outputs from the microcontroller to cut the power to the laser diodes and the image sensors when they are unused. Specifically, to cut the power to the image sensor, the power wire of the USB 3.0 cable of the image sensor is cut and a MOSFET-based digital switch is inserted into the power line.
5. Control program: A Windows application written in C# with a graphical user interface (GUI) enables a user to initiate the screening of the current sample in addition to various other functionalities, such as customizing image acquisition parameters, performing a live view of the diffraction patterns, taking a snapshot, and stopping the acquisition.

### Image acquisition

After the sample is loaded onto the device and has settled for a waiting period of 3–4 min (4 min for lysed whole blood and 3 min for artificial CSF; refer to Fig. 2 for details), the user presses the “record” button on the GUI to start acquisition. During screening, our device is programmed to scan the capillary tubes at 36 discrete positions, with a distance of ~4.7 mm between spatially adjacent positions. This approach yields a total screening volume of 36 (discrete scanning positions) × 29.4 mm^2^ (FOV of the image sensor) × 1 mm (channel height of the capillary tube) ≈ 1.06 mL per lensless speckle imager, and ~3.18 mL for the three parallel imagers combined. At each of the 36 positions, to achieve a high frame rate (~26.6 fps), the image sensor’s FOV is split into two halves (i.e., the upper 1374 rows of the pixels and the lower 1374 rows of the pixels). Each half sequentially captures 61 frames (for lysed blood) or 41 frames (for CSF) (refer to Supplementary Methods and Supplementary Fig. S4).

The image sensor’s temperature rises when it is powered, leading to temperature gradient-induced convection flow of the liquid sample (refer to Supplementary Methods and Supplementary Fig. S5). To mitigate these problems, two measures are taken. First, instead of unidirectionally scanning the 36 positions, the device scans in a back-and-forth manner (refer to Supplementary Movie 1). Let the 36 positions be represented by positions #1, #2, …, #36, which are spatially ordered. Instead of scanning in the order #1, #2, …, #36, we programmed the device to scan with a larger step size of 9 positions. Whenever the scanning head cannot move forward with this step size, it returns to the unscanned position with the smallest position number. That is, the device first scans positions #1, #10, #19, and #28. The scanning head returns to position #2, followed by #11, #20, and #29, and so on. This scanning pattern largely prevents heat accumulation at a given section of the capillary tube, which has sufficient time to cool down before the scanning head returns to its vicinity. As a second measure, a 6 s “downtime” is added between scanning positions to enable the image sensor to cool down. After completing the acquisition at a given position, the power to the image sensor is cut by a MOSFET-based digital switch added to the USB 3.0 cable. After a 6 s wait time, the stepper moves the scanning head to the next position, where the power to the image sensor is restored to capture the next set of images.

During our testing, we save the acquired images to a hard drive for processing. All three image sensors, which capture uncompressed 8-bit images, generate a total data rate of ~421 MB/s, which slightly exceeds the average write-speed of our solid-state drive (SSD). Therefore, a queue is created in the random-access memory (RAM) for each image sensor to temporarily buffer the incoming image data, and another thread is created to constantly move the image data from the buffer into the SSD. Because all remaining image data can be fully saved to the SSD during the previously mentioned downtime between two positions, the total image acquisition time per test is not increased due to the limited write-speed. As a more time-efficient alternative, the acquired images can be temporarily stored in the RAM while they are constantly moved to the GPUs for processing in batches that correspond to each image sequence. In this way, the image processing can be concurrently performed with the image acquisition, which reduces the total time per test (refer to the Results section, Fig. 2, and Table 1).

### Image processing using CMA and deep learning-based identification

We developed CMA to generate 3D contrast from particle locomotion in noisy holograms and speckled interference patterns and applied deep learning-based classification to identify the signals that correspond to the parasite of interest. As an example, Fig. 3 depicts the procedure that we used to detect trypanosomes from lysed whole blood, whereas in other application settings (e.g., trypanosome detection in CSF), we applied minor changes to the procedure, as detailed in this section. The algorithm takes the raw holographic diffraction patterns acquired by each image sensor at each scanning position as input. We denote **A**_*i*_ (*i* = 1, …, *N*_F_) as the raw frames, where *N*_F_ is the total number of recorded frames in each sequence. The algorithm consists of the following steps:

1. Hologram preprocessing to mitigate the variations and nonuniformity of the illumination

Every 8-bit raw image acquired by each image sensor (Fig. 3a) is divided by a “background” intensity pattern that represents the nonuniformity of the laser diode illumination source, which was previously computed from Gaussian-smoothed and averaged raw images in a negative control experiment and stored to be applied by other experiments. The hologram is further normalized (divided) by its own mean value, which yields the illumination-corrected holograms **Ã**_*i*_ (*i* = 1, …, *N*_F_) (Fig. 3b).

2. Determining the range of axial-distances of the fluid sample under test

In the case of lysed blood, most of the cell debris tend to fully sediment within 4 min wait time (Fig. 2) and form a thin layer of particles at the bottom of the channel. We utilized this effect to our advantage by applying the “Tamura coefficient of the gradient” (ToG) autofocusing criterion^54,55^ to automatically determine the z-distance of the bottom of the fluid sample (Fig. 3c), which is denoted as *z*_b_. We defined our range of digital z-scanning as [*z*_b_ − 200 μm, *z*_b_ + 1200 μm] with a 50 μm step size. In addition to the 1 mm expected channel height, we employed an extra scanning range of ±200 μm to tolerate possible errors in the channel height, tilting of the channel, and errors in autofocusing. This approach renders our hardware much less complicated and inexpensive because we do not need tight tolerances in our scanner design and sample holder placement.

In the case of clear media, such as CSF where objects/particles are sparse, autofocusing to the bottom of the channel can be challenging. Therefore, we precalibrated the *z*_b_ distance of each capillary tube (refer to Supplementary Methods), which only needs to be conducted once, and applied it throughout our experiments. Because *z*_b_ is precalibrated, i.e., not adaptively calculated for each sample, we specify a larger range of digital z-scanning, [*z*_b_ − 500 μm, *z*_b_ + 1500 μm], also with a 50 μm step size. Note that *z*_b_ slightly differs for each of the three channels of the device.

3. CMA algorithm to generate contrast from locomotion

We denote the z-distances to be scanned as *z*_*j*_ (*j* = 1, …, *N*_z_), as determined by the previous step. We digitally propagate each element of **Ã**_*i*_ to each element of *z*_*j*_ with a high-pass filtered coherent transfer function (Fig. 3d); refer to Supplementary Methods for details) to obtain

1 $${\bf{B}}_{i,j} = {\text{HP}}\;[{\text{P}}(\widetilde {\bf{A}}_i,z_j)]$$

where P represents the angular spectrum-based back-propagation^56–58^, HP represents high-pass filtering, and *i* = 1, …, *N*_F_, *j* = 1, …, *N*_z_ .

Next, a time-averaged differential analysis with OFN is applied (Fig. 3e):

2 $${\bf{C}}_j = \frac{1}{{N_{\text{F}} - \delta _{\text{F}}}}\mathop {\sum}\limits_{i = 1}^{N_{\text{F}} - \delta _{\text{F}}} {\frac{{\left| {{\bf{B}}_{i + \delta _{\text{F}},j} - {\bf{B}}_{i,j}} \right|}}{{\exp \left[ {\gamma \cdot \frac{1}{2}\left| {{\bf{B}}_{i + \delta _{\text{F}},j} + {\bf{B}}_{i,j}} \right|} \right]}}}$$

where *δ*_*F*_ is the subtraction frame interval, $\exp \left[ {\gamma \cdot \frac{1}{2}\left| {{\bf{B}}_{i + \delta _{\text{F}},j} + {\bf{B}}_{i,j}} \right|} \right]$ is the OFN factor, and *γ* is a parameter related to OFN that is respectively tuned for the lysed blood (*γ* = 2) and CSF experiments (*γ* = 3). Time-averaging significantly improves the SNR by smoothing out random image noise and random motion of unwanted particles/objects while preserving the true signals of motile microorganisms. OFN further suppresses potential false positive signals produced from, e.g., strongly scattering and unwanted particles/objects such as cell debris (refer to Supplementary Methods and Supplementary Figs. S6 and S7). The result of this step, **C**_*j*_, is a three-dimensional image stack.

4. Post-image processing and segmentation

The z-stack **C**_*j*_ (*j* = 1, …, *N*_*z*_) suffers from a low-spatial-frequency background that is primarily attributed to high-frequency noise in the raw images, which remains when performing high-pass filtered back-propagation and frame subtraction. As shown in Fig. 3f-h, the 3D z-stack **C**_*j*_ is high-pass filtered in the z-direction by a mean-subtracted Gaussian kernel (*σ*_*z*_ = 250 μm) and the negative pixels are clipped to zero, which yields **D**_*j*_ (*j* = 1, …, *N*_z_). **D**_*j*_ is projected onto a 2D image **E** using maximum intensity projection (MIP) to simplify the subsequent segmentation and classification steps. High-pass filtering is applied in 2D (mean-subtracted Gaussian kernel, *σ*_*x*_ = *σ*_*y*_ = 25 μm) to remove the residual background, and the negative pixels are clipped to zero, which yields **F**.

Segmentation of candidate signal points within **F** is performed by 2D median filtering (3 × 3 pixel window, pixel size = 1.67 μm) and thresholding (threshold = 0.01 for detecting trypanosomes in lysed blood and 0.02 for detecting trypanosomes in CSF) followed by dilation (disk-shape structuring element, radius = 2 pixels, pixel size = 1.67 μm) and searching for connected pixel regions. Connected regions that are smaller than 5 pixels are discarded. 64-by-64 pixel image patches centered around the pixel-value-weighted centroids of these connected regions are cropped from **F** (without 2D median filtering) and are used for the downstream identification by a deep learning-based classifier.

5. Deep learning-based classifier for detection of motile trypanosomes

Because the motility of trypanosomes varies within a population and trypanosomes only weakly scatter light, the signal intensities of some of the less-motile trypanosomes may fall below the “false positive” signals due to unwanted particles, even when OFN is applied. However, the mode of motion fundamentally differs between a trypanosome and an unwanted particle. An unwanted particle usually has a drifting motion due to, e.g., convection or slight tilting of the capillary tube, whereas a trypanosome typically “wiggles” with high-frequency flagellar beating and low directional velocity. These differences in motion patterns also produce differences in the typical shape of their signal spots, which create useful features that can be learned by a machine learning model for improved detection results. To exploit this opportunity, a CNN that consists of three convolution blocks followed by two fully connected layers is built and trained to identify true signal spots created by motile trypanosomes. The detailed network structure is shown in Supplementary Fig. S8, which is separately trained for trypanosome detection from lysed blood and CSF samples (refer to the Supplementary Methods for details).

6. Generation of test results

The image processing steps (Fig. 3a–j) are repeated for each captured image sequence and each image sensor. The total detected number of trypanosomes from all positions are summed and divided by the total screened volume (~3.18 mL) to calculate the detected parasitemia. For lysed blood, the parasitemia is further multiplied by a factor of 4, i.e., the dilution factor introduced by lysis, to calculate the parasitemia in the original whole blood sample.

7. 3D localization of motile microorganisms and movie generation

Our technique also offers the capability to locate the motile microorganisms in 3D and generate in-focus amplitude and phase movies of them for a close-up observation using the following steps: for each signal spot that is classified as positive by the CNN classifier, we return to the corresponding z-stack **D**_*j*_ (*j* = 1, …, *N*_z_) and only crop out a “column” that is 30 × 30 pixels in x–y, centered around this spot, while spanning the entire z-range (*N*_z_ layers). An autofocusing metric is used to evaluate each of the *N*_z_ layers, and the layer that corresponds to the maximum value of the autofocusing metric corresponds to its in-focus position. We tried ToG and Tamura coefficient-based criteria^54,55,59^; both work very well for this purpose. While the current z-localization accuracy is limited by the z-step size that we chose (Δ*z* = 50 μm), it can be further improved by finer z-sectioning. Using the currently obtained z-localization distance as an initial guess, we perform high-pass filtered back-propagation and differential analysis (detailed in Step 3) over a z-range of ±100 μm around the initial guess with a finer z-step size of 5 μm. However, OFN is disabled this time; in other words, the exponential normalization factor in Eq. 2 is removed due to OFN’s side effect of slightly weakening the signal at the optimal focus distance, where the object function of the microorganism is the strongest. Autofocusing is performed again over the same 30 × 30-pixel region over different z-layers. The previously determined x–y centroid, in addition to the newly found z-distance, is used as the 3D location of this motile microorganism. Because the additional high-pass filtered back-propagation and differential analysis may be only performed on a smaller region of interest (ROI) around each given spot (e.g., in our case, an ROI of 512 × 512 pixels is used), the 3D localization is computationally efficient. The 3D localization capability can be applied to generate movies (detailed in this section) or study microorganism behaviors in biological or biomedical research settings.

Using the obtained 3D position of each motile microorganism, a movie of each detected microorganism can be generated by digitally back-propagating (without high-pass filtering) each frame of the recorded raw image sequence **A**_*i*_ (*i* = 1, …, *N*_F_) or the illumination-corrected version **Ã**_*i*_ to the corresponding z-coordinate. The amplitude and phase channels of the back-propagated images are displayed side by side. The generated movies can potentially be employed as an additional way to confirm the detection results of this platform when trained medical personnel are available.

Although in Step 4 we projected the 3D contrast map (Fig. 3f) into a 2D contrast map (Fig. 3g) using MIP to simplify the segmentation and classification tasks. In principle, directly segmenting the 3D contrast map (Fig. 3f) is expected to be advantageous when the microorganisms are highly dense because it may mitigate underdetection issues related to microorganisms that overlap in x–y but are separated in *z*. Nevertheless, spatial overlap of microorganisms should not present a practical concern for the detection and counting of parasites because the probability of a spatial overlapping event is extremely low at low parasitemia (e.g., < 10^3^ mL^−1^). At high parasitemia, the decrease in the recovery rate due to spatial overlap can be simply corrected by calibration. Furthermore, from a diagnostic standpoint, the main challenge is to improve reliable detection (positive vs. negative) at very low parasitemia, not necessarily to obtain an exact measurement of the parasitemia. In this regard, overlapping parasites in x–y will not impact the usefulness of our platform.

### Timing of image processing algorithm

In this study, we used a laptop equipped with an Intel Core i7-6700K central processing unit (CPU) @ 4.00 GHz, 64 GB of RAM, and two Nvidia GTX 1080 GPUs for image processing. Table 1 summarizes the time required for our image processing workflow, simultaneously using a single GPU or two GPUs. We assume that the images captured by the imaging device during image acquisition are temporarily stored in the CPU RAM and are constantly moved to the GPU memory in batches corresponding to the scanning positions, where it is processed by the GPU (or GPUs). In this way, image processing can be concurrently performed during image acquisition, which reduces the time requirement per test. We mimic this situation by preloading existing data from the hard drive into the RAM of the computer before starting the timer, which provides a reasonable estimation of the time cost of our processing. Because the number of acquired images and the image processing workflow for lysed blood and CSF differ (refer to previous subsections and Supplementary Methods), their timing results are individually calculated. In Table 1, the timing results for lysed blood and CSF are separated by “/”.

## Electronic supplementary material

Supplementary Information

Supplementary Movie 1

Supplementary Movie 2

Supplementary Movie 3

Supplementary Movie 4

Supplementary Movie 5
