# Supplementary material for: Motility-based label-free detection of parasites in bodily fluids using holographic speckle analysis and deep learning
Source: Light Sci Appl. 2018 Dec 12;7:108. doi: 10.1038/s41377-018-0110-1 (PMC6290798; doi:10.1038/s41377-018-0110-1)
Supplement: Supplementary file 1 — Supplementary Information [file 41377_2018_110_MOESM1_ESM.pdf]

Supplementary Information for

Motility-based label-free detection of parasites in bodily fluids using holographic speckle analysis and deep learning

Yibo Zhang[1,2,3]†, Hatice Ceylan Koydemir[1,2,3]†, Michelle M. Shimogawa[4]†, Sener Yalcin[1], Alexander Guziak[5], Tairan Liu[1,2,3], Ilker Oguz[1], Yujia Huang[1], Bijie Bai[1], Yilin Luo[1], Yi Luo[1,2,3], Zhensong Wei[1], Hongda Wang[1,2,3], Vittorio Bianco[1], Bohan Zhang[1], Rohan Nadkarni[2], Kent Hill[3,4,6], and Aydogan Ozcan[1,2,3,7]*

[1]Electrical and Computer Engineering Department, University of California, Los Angeles, CA 90095, USA
[2]Bioengineering Department, University of California, Los Angeles, CA 90095, USA
[3]California NanoSystems Institute, University of California, Los Angeles, CA 90095, USA
[4]Department of Microbiology, Immunology, and Molecular Genetics, University of California, Los Angeles, CA 90095, USA
[5]Department of Physics and Astronomy, University of California, Los Angeles, CA 90095, USA
[6]Molecular Biology Institute, University of California, Los Angeles, CA 90095, USA
[7]Department of Surgery, David Geffen School of Medicine, University of California, Los Angeles, CA 90095, USA
*Correspondence: ozcan@ucla.edu
†Equally contributing authors

Supplementary Methods

Pre-calibration of the z-distance range

In our device, the scanning head is slightly tilted due to its own weight and the unbalanced design, i.e., the image sensors that are farther away from the linear motion shafts are slightly lower. Although we have incorporated this information into the design of the capillary tube holder, there are still slight differences (~0.2-0.3 mm) in the z_2 distances among the three parallel imaging channels. The pre-calibration step was designed to further mitigate this problem and reduce the number of digital z-scans necessary, when there are not enough particles at the bottom of the capillary tube for autofocusing. To pre-calibrate the z-distance range for each of the three channels of our device, we placed one capillary tube whose bottom outer surface was purposely made dirty. We then captured three holograms when the scanning head is at the two ends of its scanning range as well as in the middle, and autofocused to the dirty surface using the three holograms respectively[1,2]. The expected z_b in this case was calculated from the averaged autofocusing distance by adding the wall thickness of the glass capillary tube. The calibration step needs to be done ***only once***. Alternatively, the pre-calibration step can also be avoided at the cost of increasing the range of digital z-scanning, which will lead to an increase in the computation time. As another alternative, if the linear system is designed in a more balanced way to eliminate tilting (e.g., using linear motion shafts/rails on both sides of the scanning head), the pre-calibration step may also be avoided.

High-pass filtered computational back-propagation

We back-propagated the diffraction patterns to the given z-distances using the angular spectrum method[3], involving a 2D fast Fourier transform (FFT), a matrix multiplication in the spatial frequency domain with the free-space transfer function, and an inverse FFT. However, because the approximate size of the trypanosomes is known, we added a high-pass filter into the transfer function in the spatial frequency domain to suppress other noises and artifacts.

The coherent transfer function of free-space propagation is given by

$$H(f_x, f_y; z) = \begin{cases} \exp\left[j\frac{2\pi z}{\lambda}\sqrt{1-(\lambda f_x)^2-(\lambda f_y)^2} \right], & \sqrt{f_x^2+f_y^2} \le \frac{1}{\lambda} \\ 0, & \text{others} \end{cases} \tag{S1}$$

where z is the propagation distance, λ is the optical wavelength, f_x and f_y are spatial frequencies in x and y, respectively.

On top of H, we added two high-pass filters, H_1 and H_2, which are used to suppress unwanted interference patterns. H_1 is a 2D Gaussian high-pass filter, which is used to suppress the low-frequency interference patterns owing to the reflection from the various surfaces in the light path, including the protective glass of the image sensor and the various interfaces of the capillary tube loaded with fluids. H_1 is given by

$$H_1(f_x, f_y) = 1 - \exp\left[-\frac{1}{2}\sigma_1^2\left(f_x^2+f_y^2\right) \right] \tag{S2}$$

where σ_1 = 25.05 μm. H_2 is used to suppress the interference patterns caused by the unwanted grooves of the manufactured glass capillary tubes. Because the grooves are oriented along the axial direction of the capillary tubes, corresponding to the y-direction in the captured images, their energy is mostly concentrated close to the f_x axis in the spatial frequency domain. Therefore, H_2 performs high-pass filtering to f_y, which is given by

$$H_2(f_x, f_y) = 1 - \exp\left[-\frac{1}{2}\sigma_2^2 f_y^2 \right] \tag{S3}$$

where σ_2 = 116.9 μm.

The final coherent transfer function, which combines H, H_1 and H_2, is given by

$$\tilde{H}\left(f_x, f_y; z\right) = H\left(f_x, f_y; z\right) \cdot \min\left\{H_1\left(f_x, f_y\right), H_2\left(f_x, f_y\right)\right\} \quad \text{(S4)}$$

where $\min\{H_1, H_2\}$ chooses the smaller filter value from H_1 or H_2.

Optimization of the subtraction frame interval δ_F and total analyzed frames N_F in the computational motion analysis (CMA) algorithm with object function normalization (OFN)

The subtraction frame interval δ_F and total analyzed frames N_F are parameters that should be optimized for the parasite (or microorganism) to be detected. δ_F and N_F are related to the subtraction *time* interval Δt and the total analyzed *time* T through

$$\delta_F = \Delta t \cdot R \quad \text{(S5)}$$

$$N_F = T \cdot R \quad \text{(S6)}$$

where R is the frame rate of the recorded sequence (i.e., 26.6 fps in our system). By optimally choosing δ_F (or Δt), the signal from the characteristic locomotion of the microorganism of interest can be amplified with respect to the noise, which includes image sensor noise in addition to unwanted random motion of the background objects/particles in the sample. N_F (or T), on the other hand, determines the window of time-averaging. A larger N_F, in general, will result in reduction of the random background noise through averaging[4,5]; but at the same time, it can potentially also weaken the useful signal if the microorganism swims away from its original location during T due to directional motion.

We optimized δ_F and N_F for trypanosome detection by evaluating the average signal-to-noise ratio (SNR) of the processed images by CMA with OFN (corresponding to Fig. 3h), for blood and cerebrospinal fluid (CSF). Signal is defined as the maximum value of the segmented hotspot, whereas noise is defined as the average value of the background, excluding signal regions. SNR is calculated as $20 \cdot \log_{10}$(Signal/Noise) (dB). 20 hotspots were randomly chosen from one imaged field of view (FOV) of a 10^4/mL trypanosome-spiked blood experiment, and 40 hotspots were randomly chosen from a 10^4/mL trypanosome-spiked artificial CSF experiment. The SNRs were averaged for blood and CSF, respectively. We varied δ_F and N_F to observe their effects on average SNR (Fig. S4). The same set of hotspots were used consistently for average SNR calculations as we varied δ_F and N_F for either blood and CSF, respectively.

As shown in Fig. S4a-b, for lysed blood, $\delta_F = 4$ ($\Delta t = 0.15$ s) and $N_F = 61$ frames ($T = 2.29$ s) leads to the highest SNR of 35.29 dB. Fig. S4b implies that the average SNR could still increase if a larger N_F (T) is used. However, due to practical time constraints of this platform as a diagnostic tool as well as heating of the sample over time, we only recorded 61 frames per image sequence.

For CSF (Fig. S4c-d), a similar effect is observed about δ_F, which maximizes the SNR at $\delta_F = 4$. However, Fig. S4d shows that the SNR drops as a function of N_F when $N_F > 41$, and therefore, the optimal N_F is chosen as $N_F = 41$. In addition, the optimal SNR for CSF ($\delta_F = 4$, $N_F = 41$) is 45.00 dB, which is ~10 dB higher than the optimal SNR in blood. Based on these observations, we conclude that because CSF is a clear medium (as compared to the lysed blood), less averaging (i.e., a smaller N_F) is needed to achieve a low noise and high SNR. Therefore, for N_F beyond 41, the benefit in SNR from more averaging is diminished, whereas other factors that decrease SNR start to become dominant, mainly due to the gradual displacement of microorganisms from their original locations.

Object function normalization (OFN) to remove potential false positives

OFN is essential to reduce potential false positives owing to strongly scattering particles/objects within the sample (see Figs. S6 and S7 for a comparison of results with and without OFN). Under slightly time-varying illumination and drifting of the fluid, strongly scattering particles/objects such as cells that are not lysed, clumps of cell debris, spiked white blood cells (WBCs) in the CSF samples or even air bubbles can

create strong contrast (hotspots) when processed by CMA. These hotspots can resemble those created by trypanosomes, leading to false positive detections. Therefore, in order to distinguish parasites of interest (especially trypanosomes) that have *weak scattering* and *strong locomotion* from other unwanted objects that have *strong scattering* and *weak locomotion*, we use the object function itself to normalize the frame subtraction corresponding to Eq. 2 in the main text. An exponential function with a properly selected γ further selectively suppresses strongly scattering objects. For trypanosome detection in lysed blood, we chose $\gamma = 2$ based on visual judgement of the resulting distinction between "true positive" signals versus potential "false positive" signals; for trypanosome detection in CSF, $\gamma = 3$.

Construction and training of the convolutional neural network (CNN) for the identification of trypanosomes

Generation of positive images for training/validation

Positive images are manually identified from experimental data with a relatively high concentration of spiked trypanosomes. For blood, one test (i.e., one scanning experiment with three capillary tubes) with a spiked trypanosome concentration of $\sim 10^4$/mL was used (no overlap with the data reported in Fig. 5). For CSF, one test with a spiked trypanosome concentration of $\sim 10^4$/mL in artificial CSF was used, and the sample was not spiked with WBCs as was done for testing. For each bodily fluid type, the images were processed using CMA with OFN followed by post image filtering and segmentation (see Methods for details), and movies were generated for the first 4000 detected candidate spots. Two human annotators jointly viewed these movies and judged the existence of a motile trypanosome in each movie characterized by a slender body and rapid beating. The resulting label for each movie was either "positive" (with a high confidence that there existed a moving trypanosome), "negative", or "uncertain". Multiple trypanosomes that co-exist in a single video are labeled as "negative" to avoid confusing the network during training. It was much easier to annotate the movies related to CSF due to the high quality of the holographic reconstruction in the clear medium; whereas for blood, the resulting labels were mostly either "positive" or "uncertain", because it was difficult to affirm that the movie did not contain a trypanosome. After manual annotation, only those that were labeled as "positive" were kept in training/validation. The "uncertain" and "negative" were discarded. This resulted in 3697 positive images for blood and 3890 positive images for CSF. Note that the movies are solely for the purpose of labeling, and the 2D maximum intensity projection (MIP) image patches resulting from CMA are used to construct the training/validation library. The images were then randomly split into training and validation sets using a four-to-one ratio. Finally, data augmentation was performed to increase the number of training images by mirroring the images horizontally, vertically, and both horizontally and vertically, resulting in 4× larger positive training libraries for blood and CSF, respectively.

Generation of negative images for training/validation

Negative training/validation images entirely came from negative control experiments (no overlap with the data reported in Fig. 5). One negative control test was used to populate the training/validation library for blood; two negative control tests were used for CSF because of fewer "false positives" per test. When segmenting the negative images, a lower intensity threshold was used (0.008 for blood and 0.015 for CSF) to generate more images, resulting in 5834 negative images for blood and 2586 images for CSF experiments. The images were randomly split into training and validation sets using a four-to-one ratio for blood and CSF, respectively. Data augmentation was performed to the negative training libraries similarly to the positive set by mirroring the images in three different ways, resulting in a 4× enlargement of the negative training library size. In addition, to improve the robustness of the trained classifier to unseen data, we also performed augmentation by replicating the negative images and multiplying by a factor of 1.5. Thus, the total enlargement factor for the negative training libraries is 8×.

Network architecture

In this work, we constructed a CNN with three convolutional blocks and two fully connected (FC) layers (see Fig. S8). Each convolutional block consists of a convolutional layer (filter size = 5×5, stride = 1, 16 channels) followed by a rectified linear unit (ReLU) layer and a max-pooling layer (filter size = 3×3, stride = 2). The first FC layer has 128 output nodes, and the second FC layer has 2 output nodes, representing the two classes (trypanosome and non-trypanosome). The outputs are then passed through a softmax layer to generate the class probabilities. Dropout (p = 0.5) is added to the first FC layer during training. The same network architecture is separately trained to identify trypanosome signals within blood and CSF.

Network training

The CNN was implemented in TensorFlow (version 1.7.0) and Python (version 3.6.2). The convolutional kernels were initialized using a truncated normal distribution (mean = 0, standard deviation = 5.5×10^{-3}). The weights of the FC layers were initialized using the Xavier initializer[6]. All network biases were initialized as zero. The learnable parameters were optimized using the adaptive moment estimation (Adam) optimizer[7] with a learning rate of 10^{-3}. A batch size of 64 was used, and the network was trained for ten thousand iterations until converged.

CUDA acceleration of the CMA algorithm

The CMA algorithm was accelerated using CUDA C++ and was run on a laptop computer with dual Nvidia GTX 1080 graphics processing units (GPUs). The most computationally intensive mathematical operations in the CMA algorithm were fast Fourier transforms (FFTs) and inverse FFTs (IFFTs). The Nvidia CUDA Fast Fourier Transform library (cuFFT) library was used to perform these operations. Thrust library was used to perform reduction (i.e., summation of all elements) of an image, which was further used to calculate the mean value of the image for normalization. Other various basic mathematical operations on real or complex-valued images were implemented using custom-written CUDA kernel functions. The CUDA code was carefully optimized to parallelize computation, maximize efficiency and minimize GPU memory usage. For instance, when performing back-propagation of the diffraction patterns to each z-distance, the high-pass-filtered coherent transfer function (Equations S1-S4) was only calculated once per z-distance, which was reused for all the frames in the time sequence. When performing time-averaged differential analysis with OFN (Eq. 2 in the main text), only ($\delta_F + 1$) back-propagated images (i.e., $\mathbf{B}_i$) needed to be stored in the GPU memory at each given time without sacrificing performance, which reduced the GPU memory usage and made it possible to process even larger-scale problems (e.g., image sequences with more frames, or performing CMA at more z-distances) or to use lower-end GPUs with less memory.

Before performing FFTs, the raw images (vertical: 1374 pixels, horizontal: 3840 pixels) were padded to a size of 1536 × 4096 pixels. The padded pixels were assigned the mean value of the unpadded image to reduce artifacts from discontinuities. Because the new dimensions are powers of 2 and 3 ($1536 = 2^9 \times 3$ and $4096 = 2^{12}$), the FFT operation was accelerated by a factor of ~2.4× compared to without padding. After IFFT was complete, the images were cropped back to the original size for other image processing steps.

COMSOL simulation of sample heating due to the image sensor

The image sensor's temperature rises when it is turned on, creating a temperature gradient above it. Therefore, the fluid within the glass tube will gradually start to flow, also causing the particles within the glass tube to move directionally[8]. As a result, the signal of motile microorganisms generated by the CMA algorithm will weaken due to a "smearing" effect; and in the meantime, the movement of the other unwanted particles will increase the background noise and false positive detections, which is undesirable. The fluid velocity due to convection is related to the height of the fluid channel. Due to the drag force near the channel wall, a thinner channel will lead to a reduced fluid velocity at a given time after the onset of heating. However, as a tradeoff, a thinner channel also results in a reduced screening throughput.

We used COMSOL simulation to estimate the flow speed inside the channel. As shown in Fig. S5a, we created a channel (1 mm inner height, 10 mm inner width, surrounded by a silica wall with a uniform thickness of 0.67 mm) filled with water. A 6 cm section of the channel was selected as the region of interest for simulation. At the center of the channel, a CMOS image sensor (modeled as a constant heat source with a surface temperature of 313 K) was placed 1 mm below the channel's bottom surface. ~313 K was the highest temperature of the image sensor during image acquisition (see Methods), experimentally measured by an infrared camera (FLIR C2). The reference temperature (room temperature) was set to be 293 K. Non-isothermal flow was used to model the water inside the channel and the air outside the channel.

Fig. S5b-c show the result of this simulation. The maximum fluid velocity magnitude inside the channel is shown, representing a worst-case scenario. As expected, the fluid velocity increases as a function of the time after onset of heating and the channel height (see Fig. S5b). We further plotted the relation between the maximum fluid velocity and the channel height in Fig. S5c, at t = 7 s after the onset of heating, which approximately corresponds to the duration of image acquisition (there is a time gap when switching from the upper half to the lower half of the image sensor's FOV). Again, the fluid velocity at t = 7 s represents a worst-case scenario, where the fluid velocity is largest. As shown in Fig. S5c, at a channel height of 1 mm, the fluid velocity is ~2.9 μm/s. Over the duration of a single image sequence of 61 frames (~2.3 s), the displacement due to fluid flow is upper-bounded by ~6.7 μm, which is acceptable when compared with the length of the trypanosome. On the contrary, if the channel height is 2 mm, the displacement will be upper-bounded by ~28 μm, which will lead to strong smearing and reduction of the signal. As a result, the channel height in this work was chosen as 1 mm.

Supplementary Figures

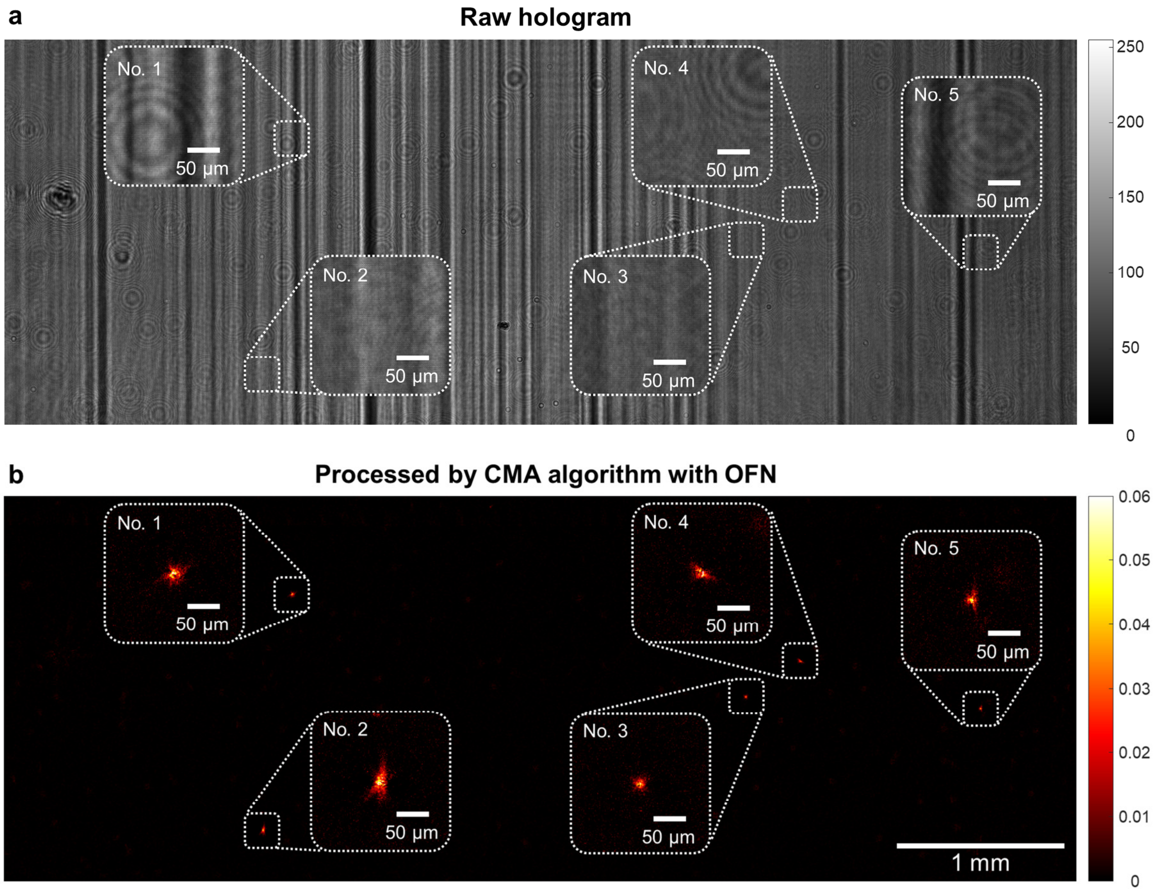

Figure S1. Imaging result of trypanosomes within artificial CSF spiked with WBCs. a One of the raw holograms in the image sequence. **b** Image that is processed by the CMA algorithm with OFN. The in-focus amplitude and phase movies of the five signal spots in this imaging FOV are shown in Supplementary Movie 3.

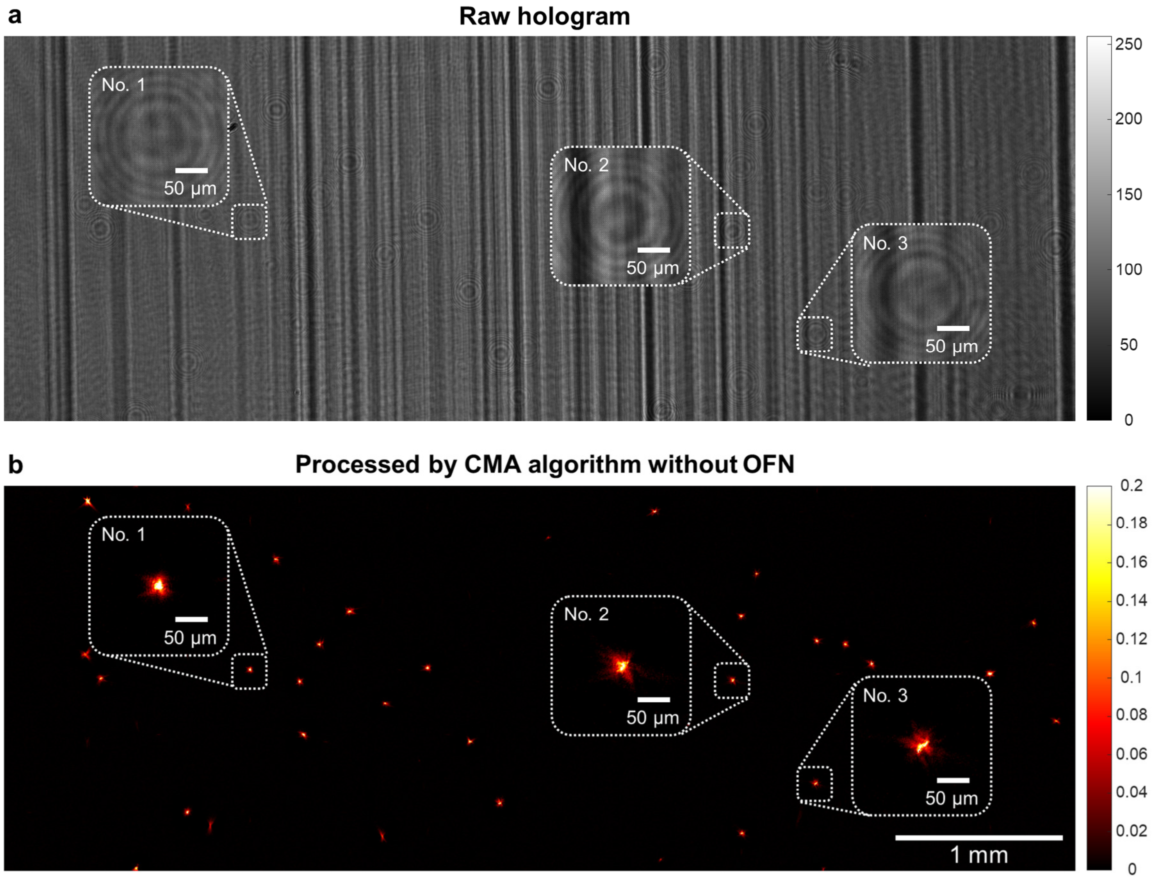

Figure S2. Imaging result of *Trichomonas vaginalis* (*T. vaginalis*) within phosphate buffered saline (PBS). a One of the raw holograms in the image sequence. **b** Image that is processed by the CMA algorithm. Because trichomonas has a strong optical phase (see Supplementary Movie 4), OFN is not applied here. The in-focus amplitude and phase movies of the three signal spots corresponding to the insets are shown in Supplementary Movie 4.

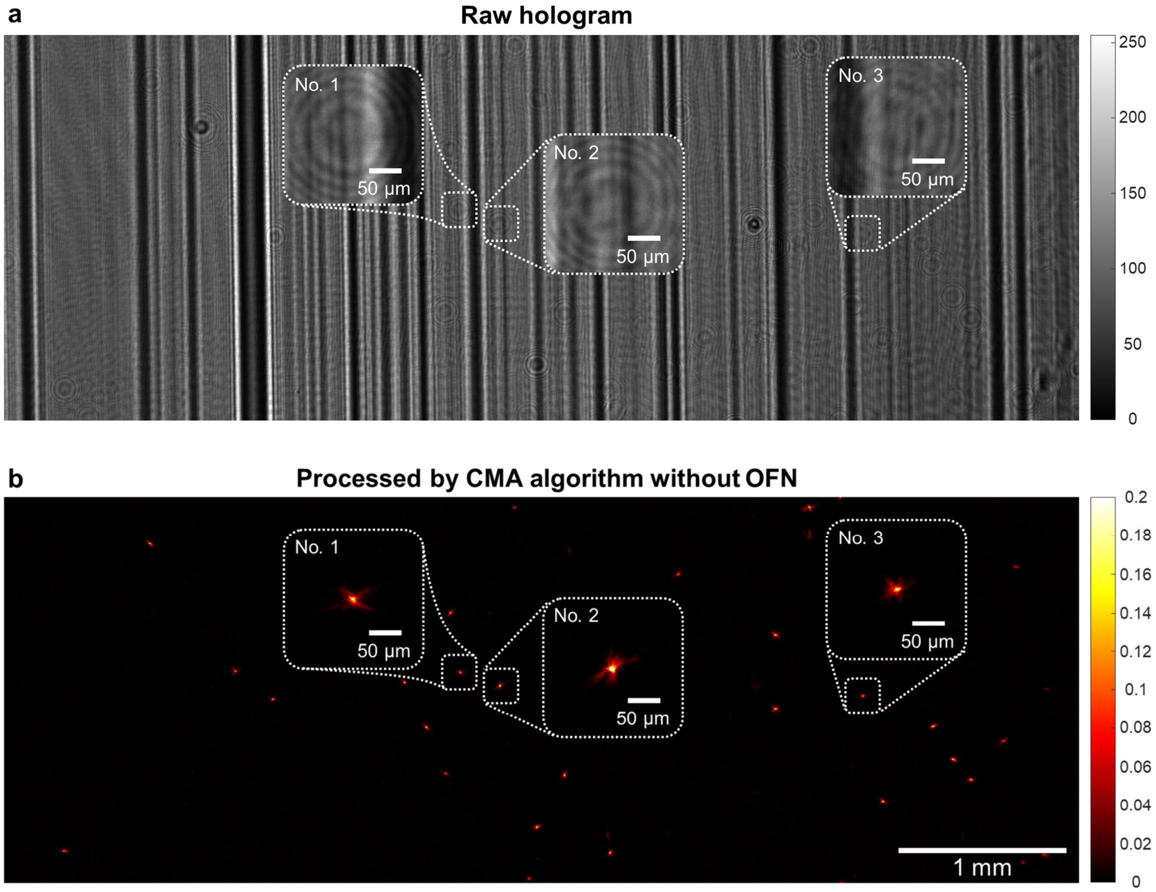

Figure S3. Imaging result of *T. vaginalis* within culture medium. a One of the raw holograms in the image sequence. **b** Image that is processed by the CMA algorithm without OFN. The in-focus amplitude and phase movies of the three signal spots corresponding to the insets are shown in Supplementary Movie 5.

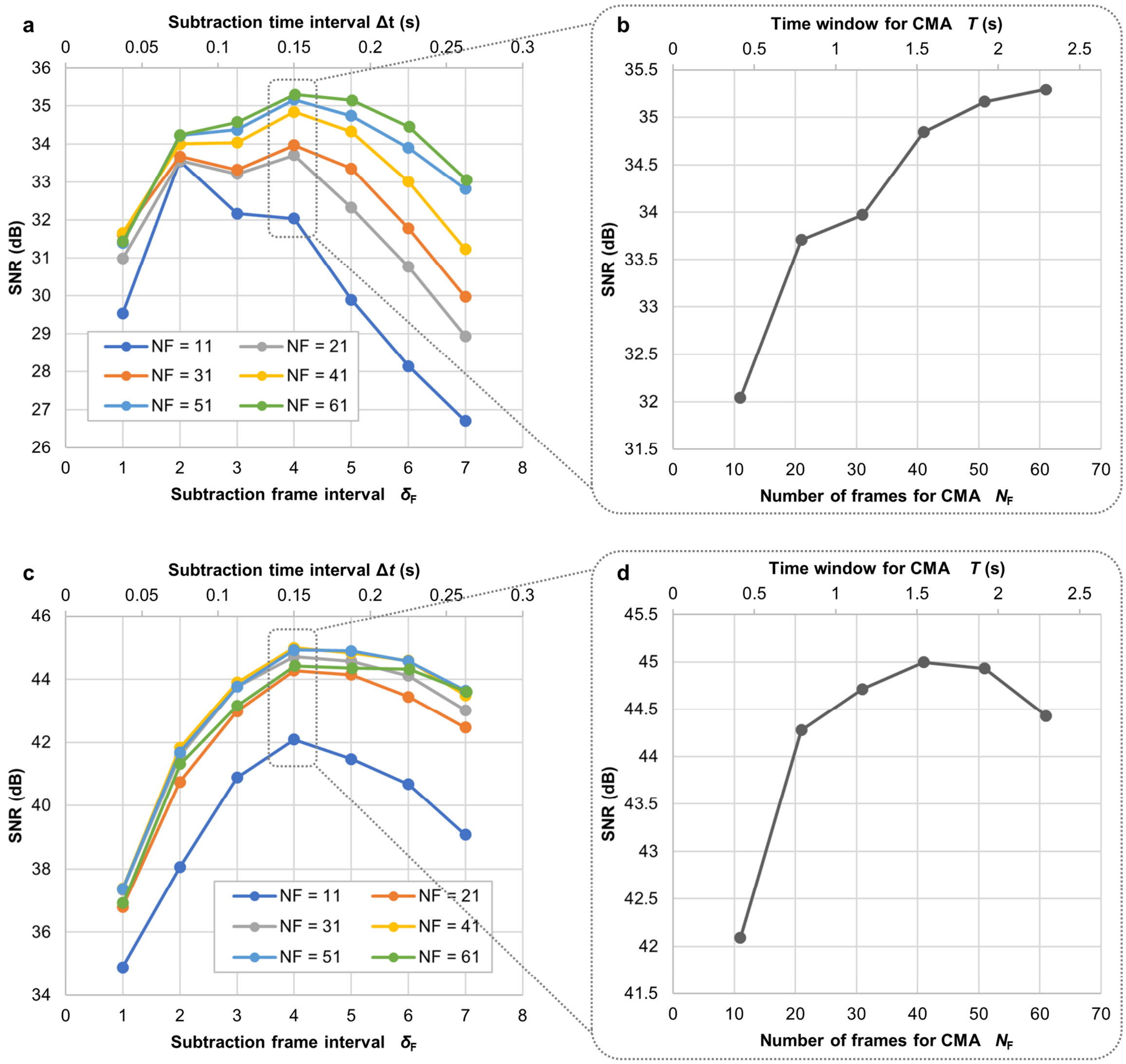

Figure S4. Optimization of the subtraction frame interval δ_F and total analyzed frames N_F for differential analysis. The SNR is used as the criterion for optimization. **a, b** Optimization for trypanosome detection in lysed blood. $\delta_F = 4$ and $N_F = 61$ leads to the optimal SNR of 35.29 dB. **c, d** Optimization for trypanosome detection in CSF. $\delta_F = 4$ and $N_F = 41$ leads to the optimal SNR of 45.00 dB.

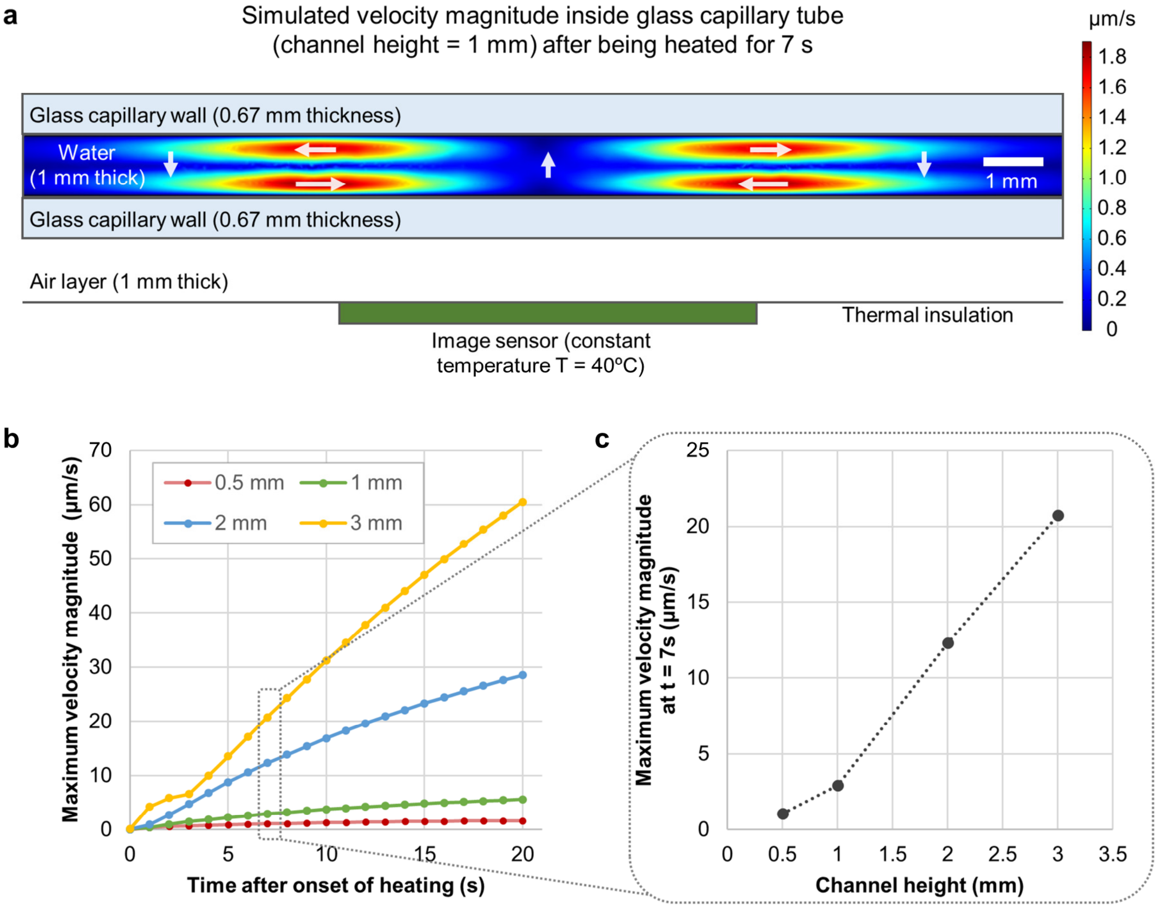

Figure S5. Simulation of the velocity field inside the glass tube due to convection after being heated by the image sensor. a Velocity magnitude distribution after the 1 mm-height glass tube is heated for 7 s (side view). The middle cross section of the glass tube is shown. **b** Maximum fluid velocity magnitude within the glass tube, as a function of time being heated. Different curves correspond to different inner height of the glass tube. **c** Maximum fluid velocity magnitude as a function of the channel height, after being heated for 7 s.

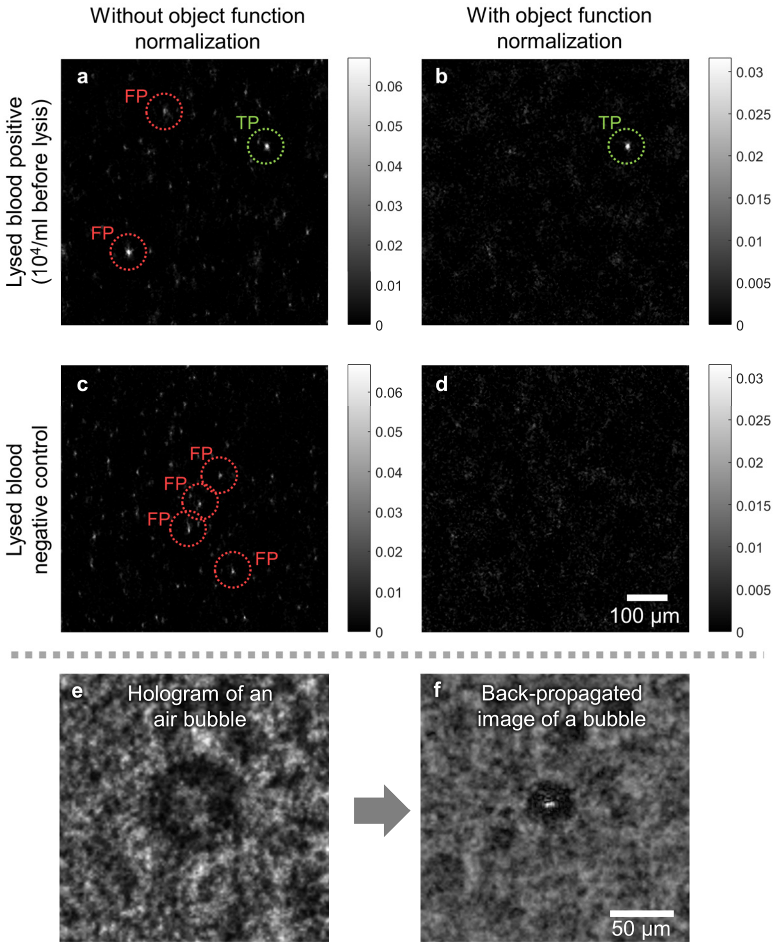

Figure S6. OFN suppresses potential "false positives" due to strongly scattering particles, demonstrated in lysed blood. TP: true positive spots corresponding to trypanosomes; FP: false positives. The false positives in the negative control sample (**c**) are suppressed after applying OFN (**d**), whereas the true positive signal is preserved ("TP" in **a** and **b**). In the positive sample, a strong false positive is created due to an air bubble ("FP" in the bottom left of **a**), whose hologram and back-propagated amplitude image are shown in **e** and **f**, respectively. It is, however, effectively eliminated by OFN, as shown in **b**.

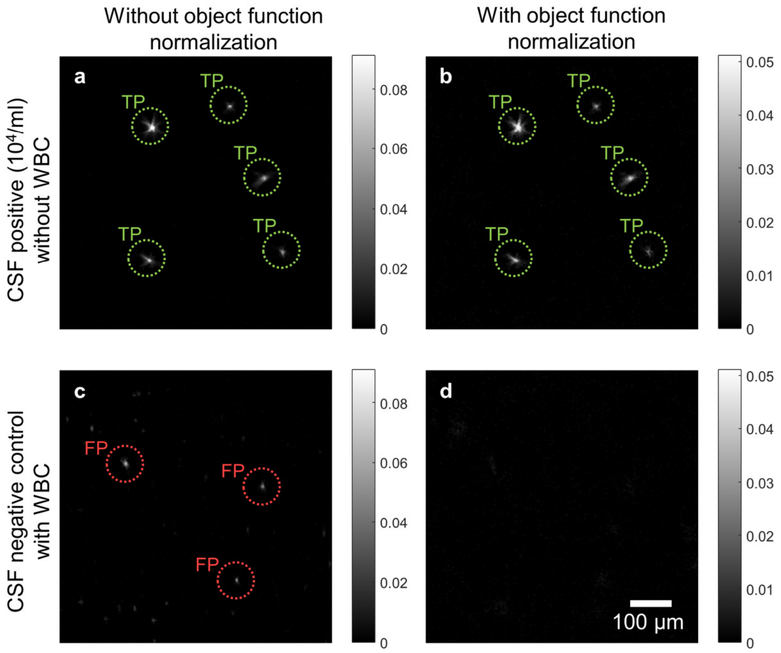

Figure S7. OFN suppresses potential "false positives" due to strongly scattering particles, demonstrated in artificial CSF. TP: true positive spots corresponding to trypanosomes; FP: false positives. The false positives due to spiked WBCs that are evident in the negative control sample (**c**) are suppressed after applying OFN (**d**), whereas the true positives are preserved in the positive sample (**a**, **b**).

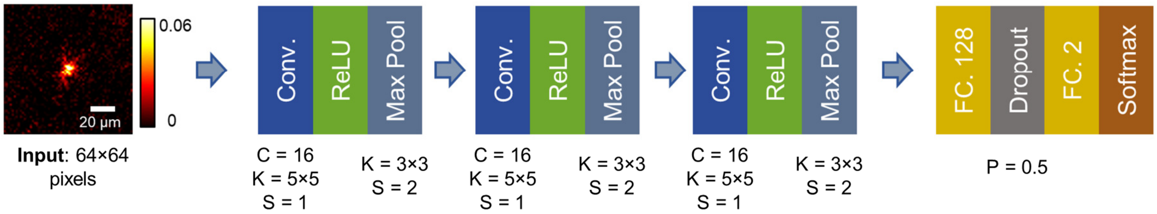

Figure S8. Structure of the CNN to automatically detect the signals created by trypanosomes. C: number of channels; K: convolutional filter size; S: stride; P: probability of dropout.

Supplementary Movies

Supplementary Movie 1: Our high-throughput bodily fluid scanning device in operation. Playback is at 16× speed.

Supplementary Movie 2: Amplitude and phase movies of three motile trypanosomes in lysed blood, corresponding to the three hotspots in Fig. 4e of the main article. Each movie is played three times.

Supplementary Movie 3: Amplitude and phase movies of five motile trypanosomes in CSF, corresponding to the five hotspots in Fig. S1. Each movie is played three times.

Supplementary Movie 4: Amplitude and phase movies of three motile *T. vaginalis* parasites in PBS, corresponding to the insets of Fig. S2. Each movie is played three times.

Supplementary Movie 5: Amplitude and phase movies of three motile *T. vaginalis* parasites in culture medium, corresponding to the insets of Fig. S3. Each movie is played three times.

Supplementary Reference

1. Zhang, Y., Wang, H., Wu, Y., Tamamitsu, M. & Ozcan, A. Edge sparsity criterion for robust holographic autofocusing. *Opt. Lett.* **42**, 3824 (2017).
2. Tamamitsu, M., Zhang, Y., Wang, H., Wu, Y. & Ozcan, A. Comparison of Gini index and Tamura coefficient for holographic autofocusing based on the edge sparsity of the complex optical wavefront. *ArXiv170808055 Physicsoptics* (2017).
3. Goodman, J. W. *Introduction to Fourier optics*. (Roberts & Co, 2005).
4. Bianco, V. *et al.* Strategies for reducing speckle noise in digital holography. *Light Sci. Appl.* **7**, (2018).
5. Bianco, V. *et al.* Quasi noise-free digital holography. *Light Sci. Appl.* **5**, e16142–e16142 (2016).
6. Glorot, X. & Bengio, Y. Understanding the difficulty of training deep feedforward neural networks. in *Proceedings of the Thirteenth International Conference on Artificial Intelligence and Statistics* 249–256 (2010).
7. Kingma, D. P. & Ba, J. Adam: A Method for Stochastic Optimization. *ArXiv14126980 Cs* (2014).
8. Kirby, B. J. *Micro- and nanoscale fluid mechanics: transport in microfluidic devices*. (Cambridge University Press, 2010).
